# Structural Similarity, Activity, and Toxicity of Mycotoxins: Combining Insights from Unsupervised and Supervised Machine Learning Algorithms

**DOI:** 10.1021/acs.jafc.4c08527

**Published:** 2025-02-27

**Authors:** Tânia F. Cova, Cláudia Ferreira, Sandra C. C. Nunes, Alberto A. C. C. Pais

**Affiliations:** Coimbra Chemistry Centre, Department of Chemistry, Institute of Molecular Sciences (IMS), Faculty of Sciences and Technology, University of Coimbra, R. Larga 2, 3004-535 Coimbra, Portugal

**Keywords:** mycotoxins, machine learning, molecular descriptors, toxicity

## Abstract

A large number of mycotoxins and related fungal metabolites have not been assessed in terms of their toxicological impacts. Current methodologies often prioritize specific target families, neglecting the complexity and presence of co-occurring compounds. This work addresses a fundamental question: Can we assess molecular similarity and predict the toxicity of mycotoxins *in silico* using a defined set of molecular descriptors? We propose a rapid nontarget screening approach for multiple classes of mycotoxins, integrating both unsupervised and supervised machine learning models, alongside molecular and physicochemical descriptors to enhance the understanding of structural similarity, activity, and toxicity. Clustering analyses identify natural clusters corresponding to the known mycotoxin families, indicating that mycotoxins belonging to the same cluster share similar molecular properties. However, topological descriptors play a significant role in distinguishing between acutely toxic and nonacutely toxic compounds. Random forest (RF) and neural networks (NN), combined with molecular descriptors, contribute to improved knowledge and predictive capability regarding mycotoxin toxicity profiles. RF allows the prediction of toxicity using data reflecting mainly structural features and performs well in the presence of descriptors reflecting biological activity. NN models prove to be more sensitive to biological activity descriptors than RF. The use of descriptors encompassing structural complexity and diversity, chirality and symmetry, connectivity, atomic charge, and polarizability, together with descriptors representing lipophilicity, absorption, and permeation of molecules, is crucial for predicting toxicity, facilitating broader toxicological evaluations.

## Graphical abstract

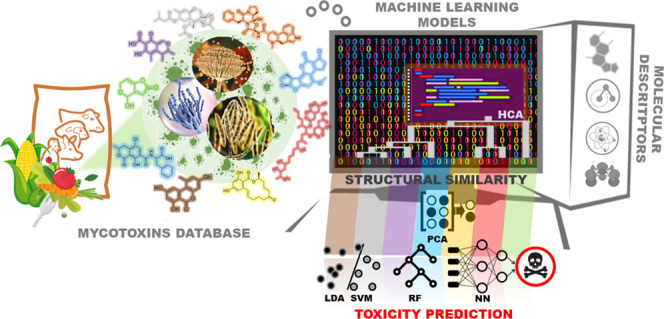

## 1 Introduction

Mycotoxins are silent fungal killers that occur in crops, water, and food, are favored by climate changes, and can cause adverse effects in humans and animals, ranging from allergic reactions to cancer and death, even at low concentrations.^1–3^ The resulting implications of cocontamination and mycotoxin poisoning worldwide are currently a trendy topic and include increased disease susceptibility, immunosuppression, impaired growth, and various cancers.^4–7^ In EU countries, mycotoxins have hampered international trading and have diverted resources toward research and regulation for reducing their effects.^8–12^

Mycotoxins are characterized by a low molecular weight, with structures ranging from single heterocyclic rings with molecular weights of scarcely 50 Da, to groups of irregularly arranged 6- or 8-membered rings with total molecular weights exceeding 500 Da, constituting a toxically and chemically heterogeneous group.^13,14^ Of these biologically active metabolites, some of which have received attention in the scientific community, are aflatoxins, deoxynivalenol, citrinin (CTN), trichothecenes, fumonisins, zearalenone, T-2 toxin, ochratoxins, patulin (PATL) and certain ergot alkaloids, due to their sociological and agroeconomic impact.^15–18^ However, only a few are well characterized and have respective effects upon humans or animals duly established. Additionally, there is little information on the interaction between concomitantly occurring mycotoxins and the consequence of toxicity.^19–24^

The most well-known mycotoxin families and their major characteristics, with one example for each family, are summarized in Table S1.^3,4,13,25–49^

The significance of mycotoxins is based on their frequency of occurrence and on the severity of the disease (mycotoxicosis) they produce in higher vertebrates.^50^ The latter can be as diverse as the chemical structures of the compounds themselves.^51^ Mycotoxicosis can be categorized as chronic or acute. Acute toxicity is characterized by a short acting time and an obvious toxic response (e.g., deterioration of liver and kidney function), while chronic toxicity is characterized by a low-dose exposure over a long period of time, often resulting in irreversible effects and cancers.^51,52^ Furthermore, some mycotoxins can affect DNA replication, producing mutagenic or teratogenic effects.^53–56^

Mycotoxin effects might be evident only years after ingestion, compromising food safety, and can only be determined by direct analysis of the toxic compound.^57–59^ ^,^ ^61^

Current research is increasingly focusing on *in silico* methods for rationalizing and predicting the occurrence and toxicity of these toxins and their degradation and transformation resulting from biological/chemical processes.^62–70^

However, to our knowledge, the use of machine learning (ML) to establish a computational framework for dealing with multidimensional data related to mycotoxins is still limited.^71–74^

The application of ML to mycotoxin-related phenomena has been focused on field contamination, based on meteorological, environmental, and agricultural data that reflects the growth and germination of fungi and, therefore, the production of mycotoxins.^71,75^

The first study where ML was applied to mycotoxins was conducted by Torelli and co-workers^75^ in a two-year study where an artificial neural network (NN) model was developed to predict fumonisins, deoxynivalenol, and zearalenone contamination of maize at the harvest time, using seven cropping system variables (Food and Agriculture Organization class, sowing and harvest dates, crop duration, kernel moisture, European corn borer treatment, and irrigation). These authors showed for the first time the potential of ML in the study of mycotoxins, emphasizing the importance of new approaches for rapid cataloging of grain lots.^75^ More recently, Camardo Leggieri et al.^71^ recorded the occurrence of aflatoxin B1 and fumonisins in maize fields and collected the corresponding cropping data over the years 2005–2018 in northern Italy. The authors built two deep neural network (DNN) models to predict, at harvest, which maize fields were contaminated with those mycotoxins, obtaining two robust models and better results when compared to AFLA-maize and FER-maize.^71^ Note that, the aforementioned models (AFLA-maize and FER-maize) both use meteorological data as input variables to predict the risk of contamination of aflatoxin B1 and fumonisins above legal limits, but these models do not use a ML approach, being considered mechanistic models.^76,77^

ML approaches have also provided information on the toxicokinetics and toxicity of some less studied mycotoxins in both food and feed. For instance, the oral toxicity and other toxicological end points of identified mycotoxins have been predicted by using the ProTox-II Web server.^78,79^ Liquid chromatography coupled to hybrid quadrupole time-of-flight mass spectrometry (LC/Q-TOF MS) and *in silico* models have been combined to determine the mycotoxin occurrence in edible tissues of Atlantic salmon (*Salmo salar*) and to predict the potential toxicity of the identified mycotoxins.^79^

The metabolomics profile of some mycotoxins (e.g., ZEA, α-ZEL, and β-ZEL) has been also explored for predicting their toxic effects using MetaTox, SwissADME, and PASS softwares.^80^

In the present study, supervised and unsupervised learning models are used in a complementary manner to accelerate the assessment of mycotoxin-caused hazards and to infer relevant relationships between their structure, family, and toxicity. Mycotoxins are first classified into groups based on their molecular structure and physicochemical properties so as to investigate their respective diversity and activity. The higher the structural similarity of the compounds, the more likely they are to share common properties and similar toxicity potency. We investigated whether the acute toxicity of these compounds can be described by combining unsupervised and supervised ML algorithms across different combinations of constitutional, topological, electronic, and biological molecular descriptors.

## 2 Characterization of Mycotoxins by Machine Learning Methods

The general procedure for addressing the characteristics and effect of mycotoxins comprises five main steps: (1) construction of chemical structures and extraction of the corresponding 3D coordinates, (2) assignment of toxicity class to each molecule using the *Globally Harmonized System of Classification and Labeling of Chemicals* (GHS), (3) calculation of constitutional, hybrid, topological, electronic, and geometric molecular descriptors for each molecule using the Chemistry Development Kit (CDK), (4) evaluation of molecular similarity based on the descriptors calculated in step (3), and (5) construction and evaluation of classification models for predicting toxicity using different combinations of molecular descriptors. Each of the five steps is briefly presented below; further details can be found in the Supporting Information.

The combination of unsupervised and supervised ML techniques supports a faster screening of mycotoxin features to predict toxicity. Hierarchical cluster analysis (HCA) and principal component analysis (PCA) were used to estimate the relative similarities between mycotoxins and their families, and supervised learning models [linear discriminant analysis (LDA), random forest (RF), support vector machines (SVM), and neural networks (NN) ] were developed to better understand the role of the descriptors selected from PCA on structural similarity and toxicity profiles.^81,82^

### 2.1 Data Description and Processing

Mycotoxins were represented by molecular descriptors, i.e., numerical values describing characteristic features of the compounds, including the number of fragments and specific functional groups present in each molecule, as well as quantitative measures, such as the partition coefficient (log *P*) and abstract attributes extracted from the graphical representation of each molecule.

A data set composed of 30 mycotoxins belonging to the better-known families was considered as a reference data set to capture the key information on the compound collection and to compare and evaluate the diversity of molecular structures and properties. A larger data set was created to include the 30 reference mycotoxins and the other 29 not-so-well-known mycotoxins from the literature. The complete list of mycotoxins is shown in Figure 1 with the code and toxicity class (not acutely toxic −0, acutely toxic −1) assigned to each mycotoxin.

**Figure 1:**
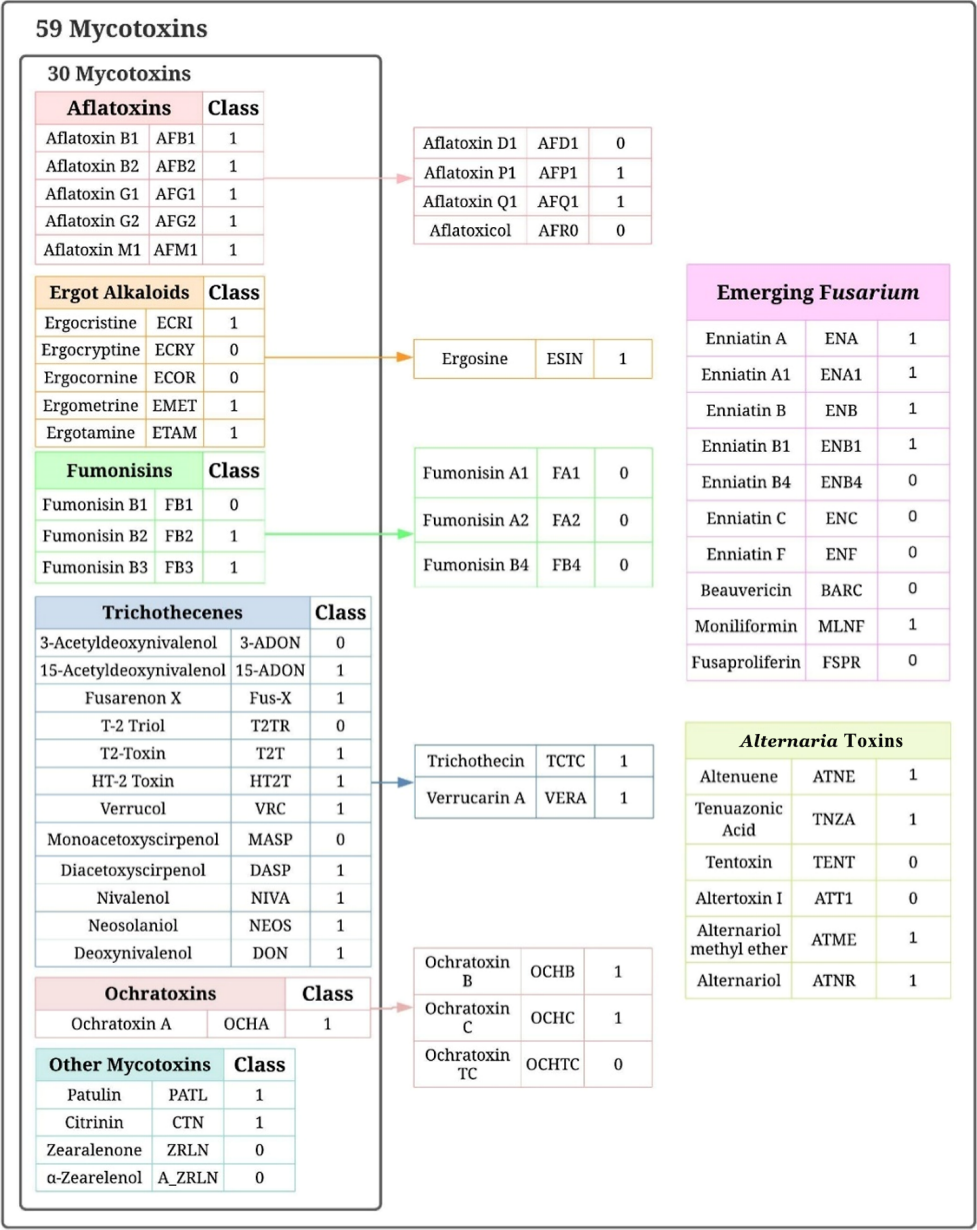
Schematic representation of the list of 30 mycotoxins included in the reference data set (left), and the 29 not-so-well-known mycotoxins (right), including their respective acronym and toxicity class (0 and 1 for not acutely toxic and acutely toxic, respectively). Of the latter, the ones signaled by the arrows have been introduced in existing families, while those positioned on the right have formed two new categories.

The Avogadro software,^81^ version 1.2, was used to draw, display, and characterize the chemical structures of mycotoxins. The molecules were then saved as Simplified Molecular Input Line Entry System (SMILES) and structural data file (SDF) formats and were imported and processed in R (version 3.6.3) and Python (version 3.8).

The two sets of mycotoxins were evaluated based on their molecular descriptors and fingerprints using ML, in the R and Python programing languages, and their respective integrating development environments, RStudio and Spider.^1,2^ The Chemistry Development Kit (CDK),^2,3^ an open-source Java framework for structural chemistry and bioinformatics that includes the package rcdk,^2–5^ was used in this study to analyze available chemical data and structures, calculate molecular descriptors, and identify and evaluate relevant molecular fingerprints.

### 2.2 Toxicity Class

There are some experimental studies^60,83–94^ reporting toxicity values for mycotoxins in rats, pigs, chickens, and other animals. However, there is not a representative number of studies using the same animal model, route of administration, conditions, and time required to establish an ML model for predicting toxicity. This limitation was circumvented by classifying mycotoxins as acutely toxic (designated as “1”) and nonacutely toxic (designated as “0”) according to the *Global Harmonized System* (GHS) classification. If a mycotoxin was previously classified as acutely toxic by the GHS, then that mycotoxin would be assigned 1. If not, the mycotoxin would be assigned 0. This classification is based on existing GHS categorizations, which involve specific limits for various forms of contamination (oral, dermal, gaseous, etc.), primarily derived from experimental data. However, for mycotoxins lacking GHS labels, it is implied that they were not subjected to direct evaluation for acute toxicity within the context of the GHS classification system. In these cases, when mycotoxin specific experimental data are not available, the GHS employs a series of steps that include intensive research on similar molecules, symptoms, animal species, and also multiple expert judgments (for details, see the eighth revised edition of the GHS, available at https://unece.org/ghs-rev8-2019).

### 2.3 Molecular Descriptors

Molecular descriptors consist of numerical features calculated for selected molecular structures.^95,96^ These descriptors were classified according to their dimensionality, depending on the molecular representations from which they were calculated.^95,96^ One-dimensional (1D) descriptors include bulk properties and physiochemical parameters such as the number of atoms, bonds, or fragments, molecular weight, and the sum of atomic properties.^95,96^ The most common type of descriptors reported in the literature are two-dimensional (2D) molecular descriptors and include molecular profiles, topological indices, and autocorrelation descriptors.^95,96^ Table S2 summarizes the descriptors used in this study.

The rcdk package^97,98^ allowed the extraction of 287 molecular descriptors, which were divided into constitutional, hybrid, topological, electronic, and geometric types. Subsequently, the computed descriptors were subjected to feature selection, which consisted of (i) removing missing values, (ii) removing highly correlated descriptors (ρ ≥ 0.6), and (iii) removing descriptors with zero variance, and were reduced to 15 molecular descriptors for the reference data set and 28 molecular descriptors for the larger data set (see Figure 2).

**Figure 2:**
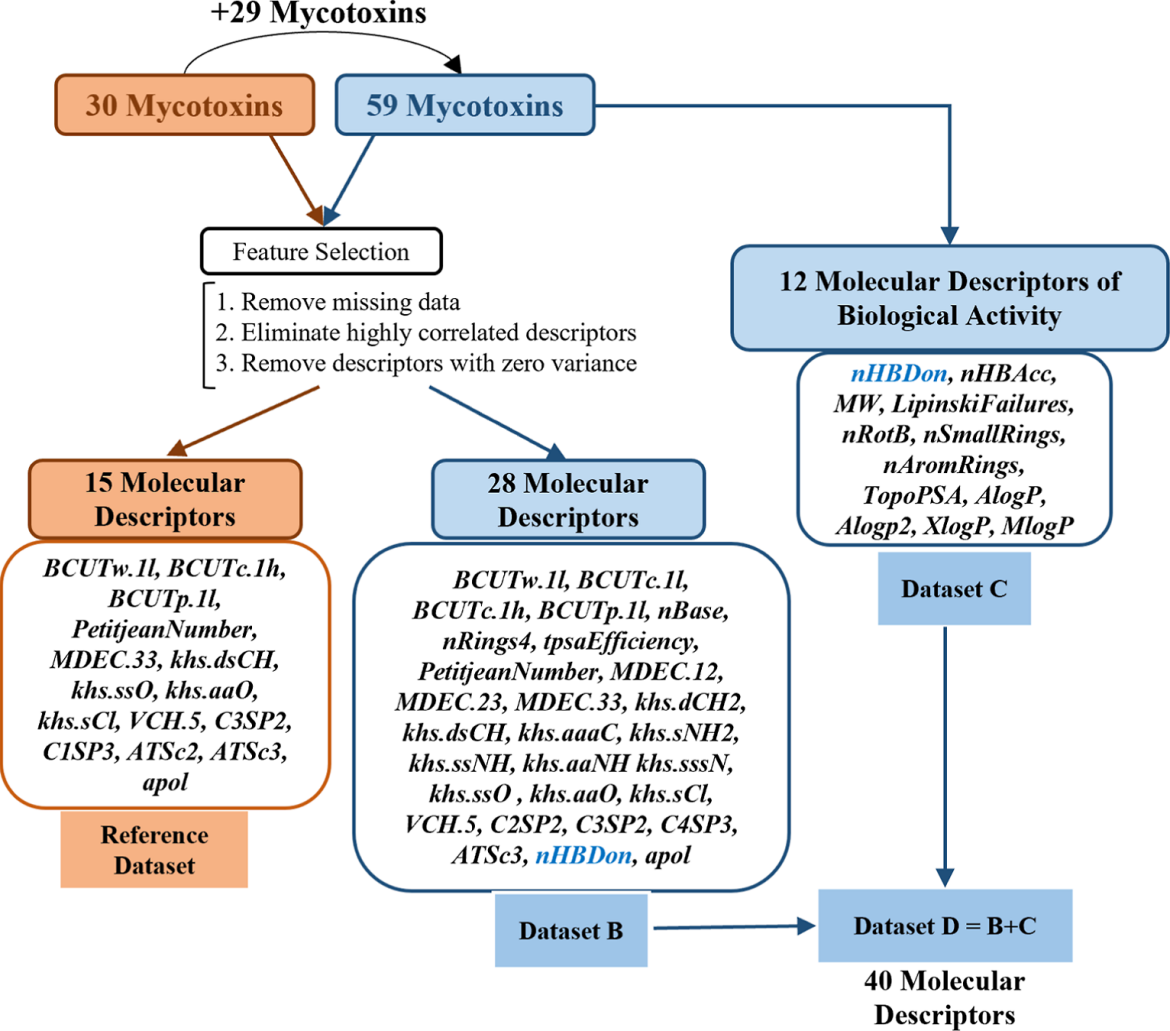
Diagram illustrating the process of selecting molecular descriptors for mycotoxin analysis. The mycotoxins are initially divided into two sets (the reference data set with 30 mycotoxins and the extended data set with 59 mycotoxins). Feature selection criteria are then applied, resulting in two subsets of molecular descriptors (15 and 28 descriptors, for the reference data set and data set B, respectively). Data set D is formed by combining descriptors from subsets B and C, yielding a total of 40 molecular descriptors for distinguishing between the mycotoxins.

Since the main objective of this work is to relate the structures and physicochemical properties of mycotoxins to their toxicity, an additional data set was also created containing 12 molecular descriptors that consider the likelihood of absorption and permeation and may help discriminate toxic compounds. Some of these descriptors fall under Lipinski’s rule of five (molecular weight (MW) ≤ 500 Da, hydrogen bond donors ≤5 (HBDon, the total number of nitrogen–hydrogen and oxygen–hydrogen bonds), hydrogen bond acceptors ≤10 (HBAcc, all nitrogen or oxygen atoms), octanol–water partition coefficient log *P* ≤ 5 (XLogP, MlogP, ALogP, ALogp2), polar surface area (TopoPSA) ≤ 140 Å, and number of rotatable bonds (nRot) ≤ 10 (see Table S2). Figure 2 illustrates the process of creating the data sets, including the number of molecular descriptors that resulted from the feature selection. Three different data sets containing 59 mycotoxins but a different number of molecular descriptors were thus created as described in Figure 2. Data were standardized using [(μ – *X*)/σ)] for PCA, HCA, and K-means analyses. The caret package from R^97^ was used to implement LDA and SVM, while the scikit-learn package from Python was used for RF and NN.

### 2.4 Molecular Similarity and Toxicity

HCA and K-means clustering were employed for structural analysis, which allowed evaluating the molecular similarity between the selected mycotoxins and their respective families, based on the respective molecular fingerprints so that relevant properties could be predicted by the cluster map and relevant chemical patterns, within and between mycotoxin families, could be identified (see Section S1 of the Supporting Information for details). By a combination of these methods, it is possible to gain a comprehensive understanding of structural similarities and toxicity profiles of mycotoxins, enhancing the robustness and reliability of the clusters identified.

PCA over scaled data was used to provide an overview of the relative positioning of chemical compounds by summarizing the respective variation in a reduced number of principal components (PCs), with the goal of building a model based on the molecular descriptors of mycotoxins to “classify” new structures and identify target properties.

The models chosen for toxicity prediction range from conventional algorithms such as LDA and SVM to ensemble methods such as RF and more complex algorithms such as deep neural networks (DNN).

The design of the NN was based on a grid search (GS) with cross-validation so that several parameters that may affect the performance of the NN model could be tested (Section S1 and Table S3 of the Supporting Information).^95^

The performance evaluation was based on the construction of confusion matrices, which provide information about the predictions, and on the area under the receiver operating characteristics curve (AUROC), which allows conclusions to be drawn about how successful the model was in separating positive and negative classes (eqs S1–S6 of the Supporting Information).^62,83,95,98–100^

## 3 Results and Discussion

HCA and K-means were used to evaluate the molecular similarity between mycotoxins based on different sets of molecular descriptors (Figure 2). PCA was used to identify relevant molecular descriptors to distinguish mycotoxins and their families. The relationship between selected molecular descriptors and the toxicity of mycotoxins was also assessed. To do this, mycotoxins were categorized based on their acute toxicity levels. These categories served as target responses in supervised learning models, which were then used to predict the acute toxicity of mycotoxins.

### 3.1 Structural Similarity

The hierarchical clustering of the reference mycotoxins, as depicted in Figure 3a, identifies 5 natural clusters reflecting, in general, the well-known families: fumonisins, trichothecenes (excepting CTN and PATL), ergot alkaloids, aflatoxins, and the mycotoxins not belonging to a specific family.

**Figure 3:**
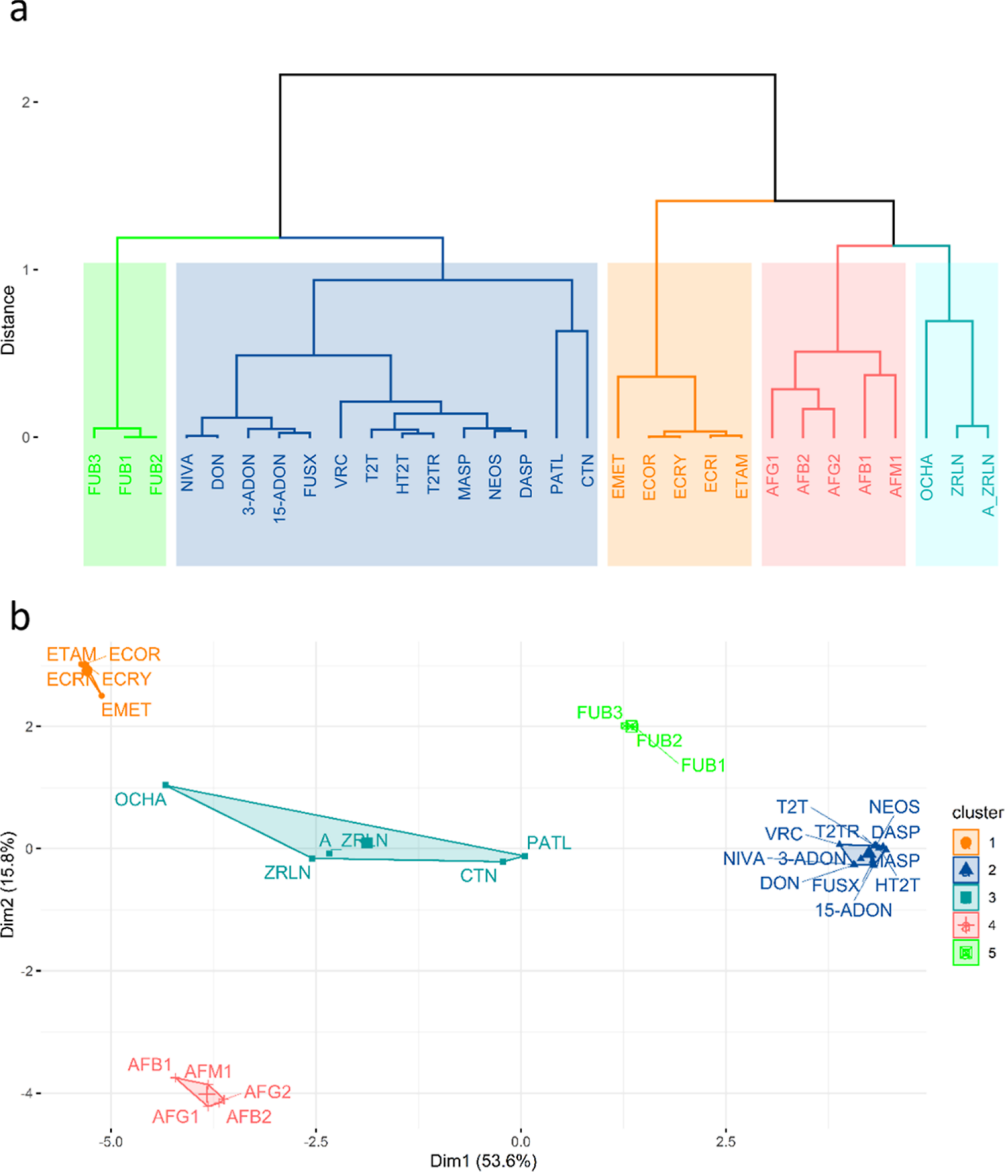
(a) Dendrogram based on Ward linkage and Euclidian distances representing the similarity between 30 mycotoxins considering 15 molecular descriptors. The different colors represent the different known families: green: fumonisins, dark blue: trichothecenes (except for CTN and PATL), orange: ergot alkaloids, red: aflatoxins, and light blue: the other mycotoxins not belonging to a specific family. (b) A clustering map obtained from the K-means clustering over the reference mycotoxins, characterized by 15 molecular descriptors.

The fumonisin group (green) is composed of fumonisins B1, B2, and B3 which are closely related metabolites that only differ in small substitutions from –OH to –H or vice versa.^101^ The trichothecene family (dark blue) is composed of type A and B trichothecenes, which are clearly separated into the 2 first subgroups of this family: on the left side are type B trichothecenes and on the right side are type A trichothecenes. These two types of trichothecenes differ in the presence or absence of a carbonyl group at the C-8 position.^102,103^ Despite PATL and CTN not being trichothecenes, they were clustered with them and included in the same subgroup. This result can be explained by the existence of bitoxigenic fungal strains with the ability to produce both PATL and CTN, which suggests some structural similarity.^104,105^ Regarding the ergot alkaloids (orange group), there is a slight separation between ergometrine (EMET) and the other ergot alkaloids, which is explained by the fact that EMET is a lysergic acid derivative, and the other mycotoxins are peptide alkaloids (also called ergopeptines) that only differ in a peptide moiety linked to the basic tetracyclic ergoline.^29^

The aflatoxin group (red) contains the most toxic known mycotoxin, aflatoxin B1 (AFB1). The characters “B” and “*G*” refer to blue and green, respectively, fluorescent colors produced by these mycotoxins under UV light. Except for aflatoxin M1 (AFM1), the selected aflatoxins are those most produced by mold metabolism. AFM1 is the hydroxylated metabolite of AFB1,^104,106–108^ corroborating their proximity in the cluster. The last cluster consists of individual mycotoxins ochratoxin A (OCHA), zearalenone (ZRLN), and α-zearalenol (A_ZRLN). A_ZRLN is the result of the animal biotransformation of ZRLN, which is consistent with its proximity. OCHA is closer to that of ZRLN and A_ZRLN than to that of the other molecules because the three mycotoxins have similar structures. These mycotoxins are frequently found together in food and feed.^109,110^

Further analysis based on K-means clustering, Figure 3b, confirms the formation of 5 distinct groups as also identified in Figure 3a, although some differences in the composition of the groups become apparent.

Figure 3b shows that PATL and CTN are clustered with OCHA, ZRLN, and A_ZRLN. The proximity between CTN and PATL is still visible in this group as well as ZRLN and A_ZRLN. OCHA, PATL, and CTN are on opposite sides of the light-blue cluster and may be at the decision boundary between their cluster and the ergot cluster (in the case of OCHA) and the fumonisin cluster (in the case of PATL and CTN).^111–114^ It can also be seen that the ergot alkaloids are farthest from the aflatoxins along the *Y* axis, while the other groups are mostly separated along the *X*-axis. Ergot alkaloids are generally more complex molecules, with higher molecular weights and partition coefficients and with more stereocenters and heavy atoms.

The differentiation of the groups along the *X*-axis can be explained by the larger structural diversity among the groups of mycotoxins. Fumonisins have long-chain hydrocarbon units, while trichothecenes are characterized by a variable number of acetoxy and hydroxyl groups, an epoxide at positions C12, and C13, and a double bond between C9 and C10.

A silhouette coefficient of 0.67 (Figure S1) validated the clustering results. Except for the group containing molecules that do not belong to any particular family (light blue), all other clusters have a silhouette coefficient close to 1, indicating that the similarity assessment and identification of the groups were performed correctly. The lower coefficient of 0.16 for the light-blue cluster is explained by the lack of cohesion within the group (Figure 3b).

Considering the larger data set of 59 mycotoxins and 28 molecular descriptors, Figure 4 demonstrates that the natural clusters identified by HCA align well with the established mycotoxin families.

**Figure 4:**
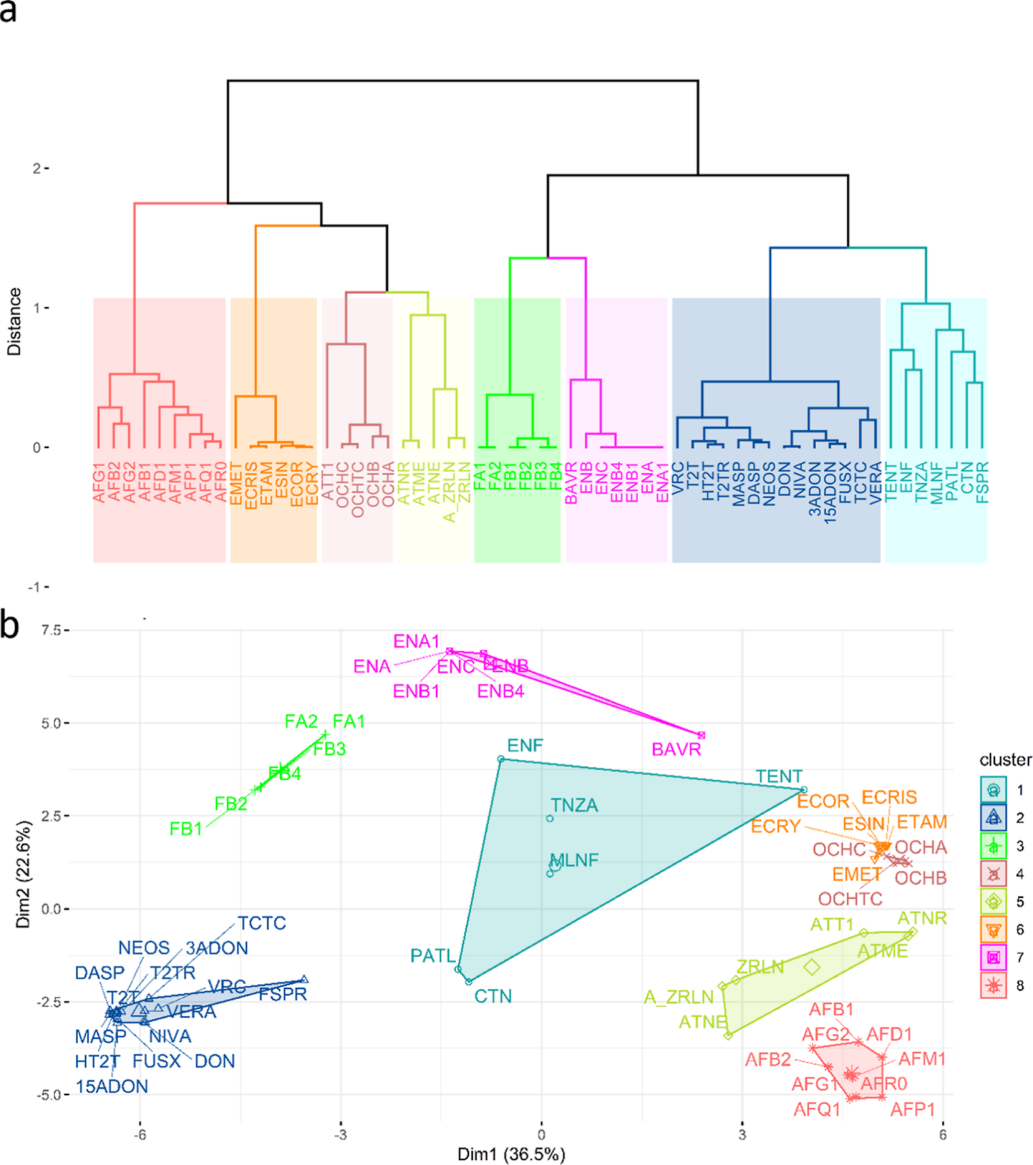
(a) Dendrogram representing the similarity structure among 59 mycotoxins considering 28 molecular descriptors, using Ward’s method over Euclidian distances. Red refers to aflatoxins, orange to ergot alkaloids, brown to ochratoxins, green to fumonisins, pink to enniatins, and dark blue to trichothecenes; light blue and yellow correspond to mycotoxins that are from different families or do not belong to a specific family. (b) The clustering map corresponding to the K-means method for the 59 mycotoxin structures, described by 28 molecular descriptors.

As far as the group of aflatoxins is concerned, with the exception of AFB1, AFB2, AFG1, and AFG2, they have been described as biotransformation products of the main metabolites in mammals.^115^ Transformation processes include oxidative hydroxylation (AFM1 and AFQ1)^116^ and O-demethylation (AFP1),^117^ justifying their proximity in this cluster. Ergosine (ESIN), also an ergot alkaloid, is positioned in the right cluster (orange) and is known to be very similar to other ergot species. A new group (brown) was formed, consisting of ochratoxins and altertoxin I (ATT1). In this group, all ochratoxins are very similar, which is in agreement with the fact that OCHB and OCHC are the dechlorinated and ethyl ester derivatives of OCHA, respectively.^118^

The yellow group consists of only 3 *Alternaria* mycotoxins, alternariol (ATNR), alternariol methyl ether (ATME), and altenuene (ATNE), and 2 other mycotoxins, ZRLN and A_ZRLN. ATNR and ATME are structurally very similar, which is why they are so close in the group.^119^

The additional fumonisin structures (FA1 and FA2) were included in the respective family but separated from the FBs because they are *N*-acetates of FB1 and FB2.^120^

The incorporation of beauvericin (BAVR) in the group of enniatins can be explained by the fact that these mycotoxins are structurally related since several *Fusarium* species can produce ENNs, differing in their amino acid residues.^121^

Compared with the reference data set, in which the group consisting of A_ZRLN, ZRLN, PATL, and CTN was identified, in the larger data set, these structures were assigned to different groups. Now CTN and PATL are grouped with moniliformin (MNLF), fusaproliferin (FSPR), and enniatins (ENF), emerging *Fusarium* mycotoxins, tentoxin (TENT), and tenuazonic acid (TNZA), which are *Alternaria* mycotoxins.

Looking at the dendrogram in Figure 4a, the light-blue cluster contains mycotoxins that are more distantly related to each other within the group than to the mycotoxins in the other clusters. As expected, the clustering map depicted in Figure 4b validates the groups identified by HCA (Figure 4a). In general, the groups are compact, except for the light-blue and green clusters, in which mycotoxins display significant structural differences.

For instance, FSPR, an emerging *Fusarium* mycotoxin, was assigned to trichothecenes, suggesting some structural similarity between FSPR and trichothecenes. This is due to the fact that mycotoxins produced by *Fusarium* species include, among others, trichothecenes and zearalenone (ZRLN).^83^

The light-blue cluster shows less cohesion because it contains mycotoxins from different families: ENF and MLNF are emerging *Fusarium* mycotoxins; TNZA and TENT are *Alternaria* mycotoxins, and CTN and PATL have not been assigned to any specific family.

The *Alternaria* toxin group also exhibits internal variability due to the structural diversity of these mycotoxins, which can be divided into 5 subclasses.^122–124^ It should be noted that ATNE, ATME, and ATNR belong to the pyranones and benzopyrones and share similar structural features, as suggested by their respective proximity in the yellow cluster.

TENT belongs to the cyclic tetrapeptides and was attributed to the light-blue cluster. In contrast with HCA, in which ATT1, belonging to perylenequinones,^125^ was included in the same cluster of ochratoxins, this molecule was attributed to the yellow cluster by K-means. These differences indicate that *Alternaria* mycotoxins may have intermediate features between those of the yellow and light-blue clusters. TENT and ENF are located at the decision boundary between the two adjacent clusters. ZRLN and A_ZRLN were grouped with certain *Alternaria* mycotoxins in the yellow cluster, and their co-occurrence has been previously reported.^126^ K-means also confirmed the molecular similarity between ATNR and ATME. These clustering results were also validated using the silhouette coefficient (Figure S2).

Further comparative analysis with existing literature demonstrates that the clustering results align with other studies that have explored mycotoxin clustering using molecular descriptors.^127–130^ For example, Tolosa et al.^127^ also identified clear clusters for fumonisins, aflatoxins, and ergot alkaloids. However, the authors emphasized toxicity-related descriptors, focusing on mutagenicity, genotoxicity, and carcinogenicity, whereas our clustering approach relied on a broader set of structural descriptors. This broader scope allows identification of overlaps within less cohesive groups, such as those containing *Alternaria* mycotoxins. In another study,^128^ the authors specifically focused on ochratoxins and used structural and enzymatic specificity descriptors to validate their clustering results. Our findings align with their results, particularly in the separation of molecules with distinct functional groups, such as ergot alkaloids and aflatoxins, demonstrating the reliability of hierarchical clustering methodologies. The chemodiversity of *Aspergillus flavus* metabolites was also explored^129^ through mass spectrometry-based clustering, allowing to distinguish clusters for chemically diverse metabolites, such as fumonisins and trichothecenes. These observations also parallel our findings and reinforce the role of structural diversity as a robust criterion for clustering. Habauzit et al.^130^ grouped enniatins and related molecules based on their biosynthetic origins and structural similarities, supporting the validity of our approach in identifying structural relationships.

### 3.2 Molecular Descriptors and Mycotoxin Discrimination

The main results of PCA for the reference data set, using the correlation matrix, are summarized in Figures 5 and 6, and in Table S4 and Figure S5. The first two principal components are able to recover ca. 47% of the data variability.

**Figure 5:**
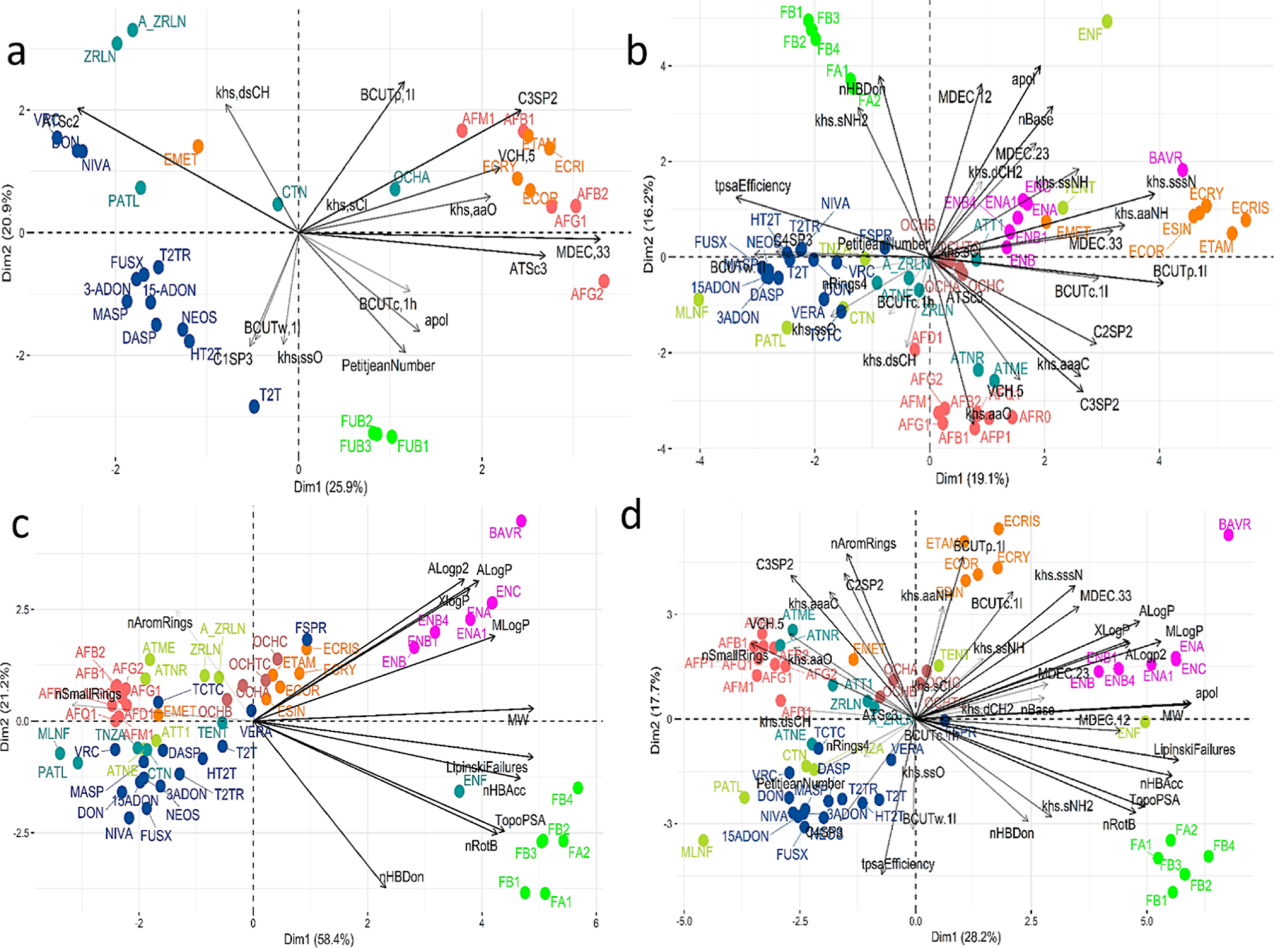
(a) Biplot representation of 30 mycotoxins (reference) described by 15 molecular descriptors on the first two principal components, recovering 46.8% of the variance. Mycotoxins are colored according to the clustering results (see Figure 3 and Supporting Information) and the molecular descriptors were selected according to feature selection method 1. (b) Biplot representation of 59 mycotoxins described by 28 molecular descriptors, (c) 12 molecular descriptors related to biological activity, and (d) 40 molecular descriptors with the addition of biological-activity-related descriptors, recovering, respectively, 35.3%, 79.5%, and 45.9% of the variance (PC1 and PC2). Mycotoxins are colored according to the clustering results (see Figure 4).

**Figure 6:**
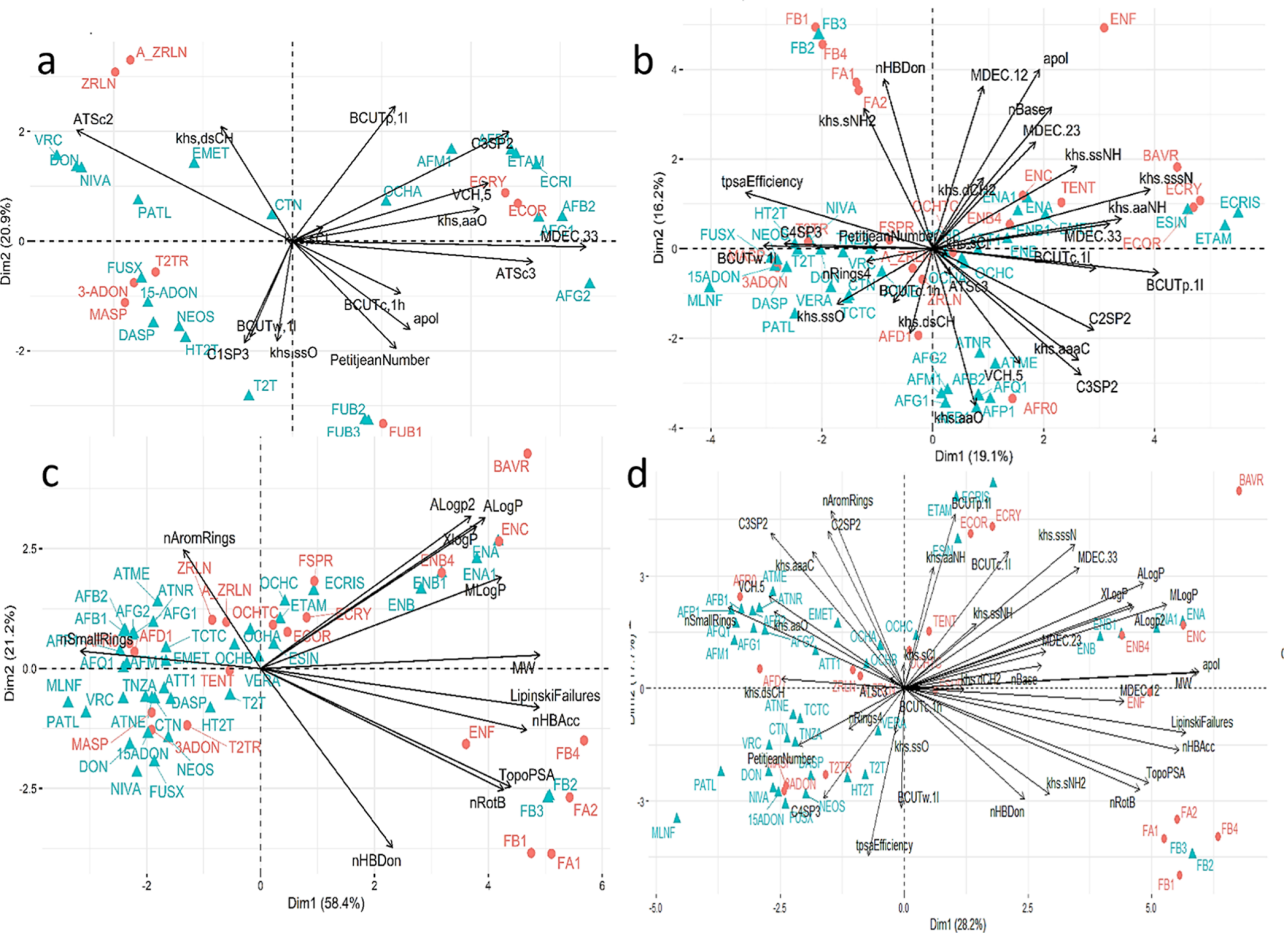
(a) Biplot representation of the reference data set containing 30 mycotoxins and 15 molecular descriptors. Biplot representation of 59 mycotoxins described by (b) 28 molecular descriptors, (c) 12 molecular descriptors related to biological activity, and (d) 40 molecular descriptors with the addition of biological-activity-related descriptors. Mycotoxins are labeled according to their toxicity (orange—not acutely toxic, blue—acutely toxic).

The molecular descriptors with the greatest influence on PC1 are MDEC.33, ATSc3, C3SP2, and ATSc2 (see Figure S3). For the second component (PC2), BCUTp.1l, khs.dsCH, ATSc2, and C3SP2 have the greatest influence. Thus, the molecular descriptors with the highest influence on PC1 are topological, while those with the highest influence on the second component are hybrid or electronic molecular descriptors (BCUTp.1l, BCUTw.1l, and apol). This observation supports the development of topological descriptors that have greater discriminatory power and better correlational ability, as described by other authors.^131^

MDEC.33, ATSc3, and C3SP2 are mainly responsible for the positioning of aflatoxins and ergot alkaloids (red and orange groups, respectively). This means that these two families share their molecular distance edge between all tertiary carbons (MDEC.33) and the autocorrelation of a Lag 3 topological structure (ATSc3), and the number of doubly bound carbons bound to three other carbons (C3SP2). ATSc2 has also a major influence on PC1 but is responsible for distinguishing some type B trichothecenes (NIVA, DON), ZRLN, and A_ZRLN.

Most trichothecenes are influenced by the descriptors BCUTw.1l and C1SP3, and the fumonisin cluster is mainly affected by the PetitjeanNumber and apol descriptors. The position of the OCHA also agrees with the clustering results, considering that this mycotoxin is close to the ergot alkaloids in both the K-means cluster plot (Figure 4b) and the PCA biplot (Figure 5a).

To infer whether there is a natural and unsupervised relationship between molecular descriptors and toxicity of mycotoxins, these structures were divided into acutely toxic and nonacutely toxic. The biplot in Figure 6a includes the toxicity class as labels for each molecule and shows that there is no direct relationship between molecular descriptors and mycotoxin toxicity in the reference data set.

To further extend the system description, PCA was applied to the larger data set, composed of 59 mycotoxins. Three different data sets were created, all with 59 mycotoxins but different numbers of molecular descriptors (see Figure 2). Data set B contains 28 molecular descriptors, Data set C contains only 12 descriptors related to biological activity, and Data set D includes all of the descriptors from both Data set B and Data set C.

The biplot for Data set B (Figure 5b) shows that mycotoxin families are in general well distinguished. The first two components represent ca. 35% of the original variability and topological descriptors dominate the higher contributions to the first two components (see Table S5 and Figure S4), except for *nBase*, apol, and *HBDon*, which are constitutional and electronic, respectively.

Aflatoxins are mainly discriminated by *khs.aaO*, which means that they possess an [*O*,*o*D_2_H_0_]­(:*):* e-state fragment. The same applies to ergot alkaloids with the descriptor khs.aaNH, which are rich in the [*N*,*n*D_2_H]­(:*):* fragment. Fumonisins are discriminated by *apol*, *MDEC.12*, *khs.dCh2*, *khs.sNH2*, and *nHBDon*.

The cluster consisting mainly of *Alternaria* toxins is widely distributed in the biplot, which is consistent with the significant structural diversity of this family. ENF is distant from its family, probably due to the apol descriptor. Additionally, BAVR might have more basic groups (nBase descriptor) or a different MDE between secondary and tertiary carbons (MDEC,23) compared to enniatines.

EMET, an ergot alkaloid, could be affected by khs.ssO or C4SP3 descriptors that distinguish this mycotoxin from other ergot alkaloids. These descriptors may also highlight structural features relevant to the trichothecenes family.

The biplot displaying the toxicity labels (Figure 6b) suggests that the discrimination based on the toxicity profile primarily occurs along the second component (PC2). The positive side of PC2 contains 25 mycotoxins, of which 52% (13 out of 25) are not acutely toxic. On the negative side, only 20% of the mycotoxins are not acutely toxic (7 out of 24).

To further explore the relationship between structural discrimination of mycotoxin families and the respective toxicity profiles, data set C was analyzed. This data set contains descriptors associated with biological activity, including nHBDon, nHBAcc, MW, XLogP, LipinskiFailures, nRotB, ALogP, MLogP, Alogp2, nSmallRings, nAromRings, and TopoPSA.

In the first component, MW, LipinskiFailures, nHBAcc, and TopoPSA are the primary contributors to the system (Table S6 and Figure S5). Conversely, for the second component, NHBDon and descriptors related to LogP play the most significant roles. The AlgoP, XlogP, and MlogP are all related to the LogP of the compounds and may exhibit high correlation. However, each descriptor captures specific chemical properties of the compounds studied, which might not be fully represented by any single descriptor alone.

The LipinskiFailures descriptor, while not a conventional descriptor in QSAR analyses, is a composite descriptor that provides a concise summary of important drug-likeness criteria, facilitating a more efficient and holistic evaluation of compound properties.^131–135^ It allows establishing a cutoff threshold that does not directly arise from the other descriptors. Although related to the other descriptors (components of Lipinski’s Rule of Five), this is an indicator that conveys specific information through the cutoff threshold, emphasizing discrimination.

Figure 5c illustrates that the distinction between mycotoxin families is not as clear as in data set B, except for the enniatins and fumonisins, which are separated along the second component. This suggests that the biological descriptors alone are insufficient to distinguish between the families, highlighting the need for topological descriptors for better differentiation. It seems that mycotoxins from the fumonisin and enniatin families are grouped together mainly due to their molecular weight, which aligns with molecular similarity findings. Specifically, AlogP influences the position of the enniatins on the positive side of PC2, while TopoPSA affects the position of the fumonisins on the negative side of PC2.

The biplot depicted in Figure 6c displays mycotoxins from data set C, each labeled according to its toxicity profile. On one side of the first principal component (PC1), there are 22 mycotoxins, while on the opposite side, there are 37. Among those on the negative side of PC1, only 8 are not considered acutely toxic, representing approximately 21.6% of the total. In contrast, about 54% (∼12) of the mycotoxins on the positive side of PC1 are not acutely toxic. Thus, the positive side of PC1 contains fewer mycotoxins overall but hosts more than double the number of mycotoxins that are not acutely toxic compared to the negative side. This observation suggests a potential correlation between the position along PC1 and the toxicity profile of the mycotoxins. These results motivate the inclusion of these biological descriptors into the previously established Data set B. As a result, a new data set, termed Data set D, was generated, consisting of 59 mycotoxins and 40 molecular descriptors. This expansion allows for a more comprehensive analysis, potentially providing deeper insights into the relationships between molecular structure, biological activity, and toxicity among the mycotoxins studied. It is evident that the biological activity descriptors, along with apol, exert the most significant influence on PC1 (Table S7 and Figure S6 in the Supporting Information). On the other hand, nAromRings emerges as the primary descriptor contributing to PC2, with subsequent contributions from BCUTp.1l, tpsaEfficiency, and the carbon species descriptors (C2SP2 and C3SP2) (see Figure 5d). Of the descriptors with the highest contributions on PC1 (Table S7 and Figure S6), only apol is not related to biological activity. While apol stands out as the descriptor with the greatest contribution, it is closely aligned with MW, LipinskiFailures, and nHBAcc (Figure 5d). On the other hand, for the second component, nAromRings emerges as the primary contributor and is associated with biological activity.

In contrast to Data set B, in Data set D, the enniatines are primarily distinguished by the biological descriptors, including MW, apol, LogPs, and LipinskiFailures (see Figure 5d). The influence of khs.sNH2 on fumonisins and khs.aaNH and BCUTp.1l on ergot alkaloids is confirmed. Additionally, the ALogP descriptor elucidates the distance of the BAVR from its family. Although aflatoxins still show influence from khs.aao, their discrimination is now also affected by the number of small rings. The descriptor C4SP3 continues to significantly influence the trichothecenes family. Ochratoxins, positioned at the origin of the biplot, do not seem to be distinctly influenced by any molecular descriptor. This observation suggests that ochratoxins might serve as reference molecules with average properties relative to the molecular descriptors given their position on the biplot. Out of the 12 descriptors related to biological activity, only two, nAromRings and nSmallRings, are situated on the negative side of PC1. Both pertain to the aromaticity of molecules, a characteristic that has been demonstrated to elevate toxicity.^134,135^ The toxicity profile depicted in Figure 6d, indicates that only 19.4% of the mycotoxins on the left side of PC1 are nonacutely toxic (7 out of 36) and 56% (13 out of 23) on the positive side.

It is noteworthy that LipinskiFailures is a descriptor influencing both absorption and permeation,^136,137^ which are important parameters in toxicity assessment. It ranks as the third highest contributor to PC1. Additionally, the biological activity descriptors played a role in positioning mycotoxins on the biplot relative to their toxicity profiles.

#### 3.2.1 Molecular Basis of Toxicity

Assessing the mycotoxins’ potential health impacts requires a distinction between acutely toxic and nonacutely toxic mycotoxins. Mycotoxins can exert their toxic effects through mechanisms such as enzyme inhibition, DNA damage, or membrane disruption, and their ability to interact with the biological targets can be strongly dependent, e.g., on the molecular conformation and stereochemistry, particularly in chiral molecules, thus impacting their toxicity.

Topological descriptors reflect how molecules are arranged and connected, shedding light on chirality, symmetry, electronic properties, and structural complexity, providing a holistic view of molecular architecture and its implications for toxicity.

Chiral molecules with asymmetric structures demonstrate unique toxicological properties, indicating that the spatial arrangement of atoms impacts a mycotoxin’s toxicity profile. Additionally, electronic properties and charge distribution within molecules play crucial roles in modulating interactions with biological targets, thereby influencing toxicity.

For instance, aflatoxin B1 (AFB1), renowned for its acute hepatotoxicity and carcinogenicity, features a distinctive difuran ring structure with a lactone moiety responsible for its toxicity. This structural motif, identified through topological descriptors related to molecular connectivity, highlights how specific functional groups contribute to acute toxicity.

Similarly, sterigmatocystin, characterized by multiple stereocenters, exhibits asymmetric structures that influence its toxicological properties, including acute hepatotoxicity and potential carcinogenicity. Topological descriptors related to chirality and symmetry, such as the Petitjean number, provided valuable insights into the relationship between molecular geometry and toxicity.

The electronic properties of a molecule, such as its electron density distribution, polarizability, and charge distribution, directly impact its reactivity and binding affinity with biological molecules. These interactions can lead to a cascade of cellular responses, ultimately determining the toxicological outcomes of exposure to mycotoxins. OCHA, known for its acute nephrotoxicity and association with renal diseases, showcases how variations in electronic properties and charge distribution reflect its toxicity profile. OCHA contains a phenylalanine moiety with a carboxylic acid group, which can undergo ionization in physiological conditions, impacting its interactions with biological macromolecules such as proteins and nucleic acids.

By influencing interactions of the molecule with biological targets, these electronic features shape OCHA’s toxicological profile of the OCHA, including its acute nephrotoxicity and association with renal diseases. Therefore, understanding these electronic properties is essential for elucidating the toxicity mechanisms of mycotoxins and designing effective strategies for risk assessment and mitigation.

Additionally, when examining Fumonisins, e.g., Fumonisin B1, which consist of a complex polyketide backbone with multiple hydroxyl and methyl groups, their structural diversity contributed to their acute hepatotoxicity and potential role in human esophageal cancer. Descriptors related to molecular diversity, such as the number of valence chains or the presence of aromatic rings, highlight the importance of structural complexity in determining toxicity.

### 3.3 Toxicity Prediction

So far, it has been shown that the biological activity reflected in some of the molecular descriptors does not contribute significantly to the differentiation of the mycotoxin families unless they are combined with very specific topological descriptors. In other words, the PCA results generally showed good discrimination between mycotoxin families; however, the relation with the toxicity of the compounds is not so clearly established. The data set exhibits a moderate and manageable class imbalance, with a 2:1 ratio of acutely toxic to nonacutely toxic mycotoxins. This imbalance could potentially bias the model toward the majority class; however, it is generally considered acceptable for most machine learning algorithms. As such, extensive corrective measures, such as oversampling or undersampling techniques, are not necessary in this case.^138–142^ To mitigate potential bias from the 2:1 class ratio, we employed a stratified 70/30 train-test split, preserving class distributions in both subsets.

#### 3.3.1 Linear Discriminant Analysis

The LDA results for each data set are summarized in Table S8. Overall, the LDA model shows overfitting for all data sets. In the reference data set, acute toxicity was successfully predicted. When the number of mycotoxins was increased, the best prediction was found for Data sets B and D, while Data set C proved to be unsuitable for prediction. It has already been noted that although Data set C has a higher variance in PCA (Table S6) and reflects important parameters of biological activity, these molecular descriptors are not sufficient to distinguish between the different mycotoxin families. This may be one reason for the poor performance of the LDA model for this data set, as it does not provide a good description of the system, especially in structural terms.

#### 3.3.2 Random Forest

The RF models indicated that Data sets B and D displayed the best performance (Table S9). Some degree of overfitting is visible but not so pronounced as for LDA.

Specificity is low in all of the data sets. RF showed that the 28 molecular descriptors in data set B were sufficient to achieve better performance. In Data set D, these 28 descriptors were combined with additional biological activity descriptors, but the results were similar.

Given the moderate class imbalance of all data sets, relying on a single metric can be misleading, and a comprehensive analysis of multiple metrics is crucial. In general, there were few false negatives as shown by the recall metric with 0 false negatives in Data sets B and D. Some false positives were present in all data sets, as shown by precision and specificity. The AUROC value indicates how well the model can distinguish acutely toxic mycotoxins from nonacutely toxic mycotoxins. In this case, there is a 70% chance (for Data sets B and D) that the model can correctly distinguish the classes.

#### 3.3.3 Support Vector Machines

The SVM results (Table S12) were very similar to those of LDA, with the reference data set giving the best results. The reference data set does not show overfitting, in contrast to the other data sets. Data sets B and D produce the same results and display overfitting. Data set C shows intermediate results, but with SVM, better results were obtained than with all other data sets containing 59 mycotoxins. Data set C promoted better results than Data set D. SVM did not prove to be a good method for predicting the acute toxicity of mycotoxins.

#### 3.3.4 Neural Networks

A neural network-based model was created following a grid search procedure that tested various parameters and yielded the combination with the best accuracy for each data set. The resulting combinations of *Gridsearch* parameters are listed in Table S11.

All data sets provided the best results for deep neural networks (DNN), the majority of which consist of three hidden layers with five or three nodes each (Data sets B and D, see Table S12). For the smaller data sets, simpler architectures with 2 hidden layers with 5 or 3 nodes each yielded the best results (reference Data set and Data set C, respectively).

In contrast to all other models, the data set consisting only of biological activity descriptors has the best performance with DNN. In all metrics, this data set showed better performance in the test set than in the training set, which is most likely due to the quality of the features since overfitting is excluded. On the other hand, this could lead us to consider underfitting, which could be explained by the narrow-hidden layers, in this particular case with only three nodes. This is due to the small overall sample and suggests that larger amounts of information could benefit from training with wider hidden layer architectures.

In summary, Table 1 shows the toxicity predictions ranked in ascending order of AUROC score, using the different models over the different data sets.

**Table 1:** Ranking of AUROC Values Obtained for the Models RF, NN, LDA, and SVM over All Datasets with Different Combinations of Molecular Descriptors^a^

model	reference	B	C	D
RF	1.00/0.40	**1.00**/**0.70**	**1.00**/**0.60**	**1.00**/**0.70**
NN	0.75/0.50	0.96/0.62	**0.65**/**0.72**	0.50/0.50
LDA	**0.89**/**0.67**	**0.95**/**0.53**	0.79/0.49	**0.98**/**0.52**
SVM	**0.75**/**0.75**	0.86/0.40	0.66/0.54	0.86/0.40

a The AUROC values refer to the training/test sets, with the best ones highlighted in bold.

LDA and SVM models performed well with the reference data set, while RF and NN models promoted better results for data sets B, D, and C, respectively. RF results for data sets B and D outperformed all other models. In general, adding the biological activity descriptors to data set B (forming Data set D) did not prove significant for improving toxicity prediction. It is noteworthy, however, that the NN model enabled the prediction of toxicity using Data set C, which contains only descriptors reflecting biological activity, with constitutional descriptors predominating (logP estimates, MW, nRotB, and Lipinski’s rule of five) and combined with a strict set of topological (TopoPSA, nSmallRings, nAromRings)^137,143^ and electronic (nHBAcc, nHBDon)^137,143^ descriptors. On the other hand, RF allowed prediction of toxicity using mainly structural information and performs well in the presence of descriptors reflecting biological activity. NN models appear to be more sensitive to biological activity descriptors than RF.

### 3.4 Concluding Remarks

A rapid and comprehensive description of the influence of molecular and physiochemical descriptors on the molecular similarity and toxicity of multiple mycotoxins and their families is proposed by combining unsupervised and supervised ML models.

The correspondence between known mycotoxin families and natural clusters identified on the basis of underlying molecular similarity patterns is determined by HCA and K-means, for which predominantly hybrid and topological descriptors determine the discrimination between the well-known mycotoxin families in structural terms. The distinction is thus made on the basis of information on (i) molecular structure and diversity (BCUTs),^134,135^ which contain contributions from all atoms (atomic charge and polarizability values and atomic H-bonding abilities) and thus reflect the topology of each mycotoxin, (ii) chirality and symmetry (PetitjeanNumber),^144^ (iii) the molecular distance edge between all tertiary carbons (MDEC.33),^145,146^ (iv) the molecular connectivity in terms of (iv) the number of −O–,:O:, and −Cl fragments (Khs.ssO, khs.aaO, khs.sCl),^145,147,148^ (v) carbon types or hybridization C2SP2 and C1SP3,^149,150^ and (vi) the presence of valence chains of order 5 (VCH-5),^143^ as well as information on (vii) the autocorrelation of topological structure descriptors, weighted by charges ATSc2 and ATSc3,^145,151^ and electronic descriptors in terms of (viii) the sum of atomic polarizabilities (apol).

PCA also suggests that topological and electronic descriptors are crucial for distinguishing mycotoxins by their families, that constitutional molecular descriptors are not sufficient to represent this information, and that the descriptors C4SP3, khs.ssO, khs.dsCH, khs.aaO, nRings4, and nSmallRings can influence the respective acute toxicity. These descriptors are relevant for predicting toxicity and need to be combined with additional BCUTs, PetitjeanNumber, and biological activity descriptors (data sets B and D) to predict mycotoxin toxicity using RF and LDA models.

In conclusion, for a comprehensive understanding of the molecular similarity and toxicity prediction of mycotoxins and other toxic compounds, it is essential to integrate information reflecting biological activity alongside constitutional, topological, and electronic descriptors. These descriptors include features such as molecular lipophilicity (e.g., MLogP), molecular weight (MW), the number of nonrotatable bonds (nRotB), and adherence to Lipinski’s rule of five. Additionally, descriptors capturing structural complexity and diversity, chirality, symmetry, connectivity, and atomic charge and polarizability play significant roles in distinguishing between different toxic compounds. By combining these diverse sets of descriptors, we can enhance our ability to discriminate between toxic compounds and predict their toxicity accurately.

## Supplementary Material



## Notes

Detailed information on mycotoxin families and chemical structures; molecular descriptors extracted from the CDK library; type of molecular fingerprints; workflow for data set creation and feature selection; parameters for performing NN; HCA, K-Means, and PCA results; contributions of molecular descriptors (eigenvalues, eigenvectors, and variance) to discrimination between mycotoxins and for toxicity prediction; R and Python packages and methods for clustering, PCA and classification; and performance evaluation metrics for the LDA, RF, SVM, and NN models (PDF)
