# Supplementary material for: Structural Similarity, Activity, and Toxicity of Mycotoxins: Combining Insights from Unsupervised and Supervised Machine Learning Algorithms
Source: J Agric Food Chem. 2025 Feb 27;73(10):6173–88. doi: 10.1021/acs.jafc.4c08527 (PMC12772517; doi:10.1021/acs.jafc.4c08527)
Supplement: Supplementary file 1 [file jf4c08527_si_001.pdf]

Supporting Information

Structural similarity, activity and toxicity of mycotoxins: combining insights from unsupervised and supervised machine learning algorithms

Tânia F. Cova*, Cláudia Ferreira, Sandra C.C. Nunes, Alberto Pais*

Coimbra Chemistry Centre, Department of Chemistry, Institute of Molecular Sciences (IMS), Faculty of Sciences and Technology, University of Coimbra, Coimbra, Portugal

* Corresponding authors: tfirmino@qui.uc.pt (T.C.), pais@qui.uc.pt (A.P.)

Contents

The supporting information contains 36 pages, 6 Equations, 14 Tables and 10 Figures

List of Equations

Equation (S1). Accuracy.

Equation (S2). Sensitivity (true positive rate, TPR).

Equation (S3). Specificity (true negative rate, TNR).

Equation (S4). Precision (positive predictive value, PPV).

Equation (S5). Negative predictive value (NPV).

Equation (S6). Area Under the Receiver-Operating Characteristics curve (AUROC).

List of Tables

Table S1. Summary of the major features of well-known mycotoxins families………………..….S7

Table S2. Summary of 43 molecular descriptors (15 for the reference data set and 28 for the larger data set) by category (hybrid, topological, electronic, constitutional), extracted from the CDK library after a standard feature selection procedure……………………………………………………..S8

Table S3. Grid search combination parameters for the NN model. Only the best performing tested models are shown.…………………………………………………………………………S12

Table S4. Eigenvalues and data recovery evolution with respect to the number of principal components for the reference dataset composed by 30 mycotoxins and 15 molecular descriptors. The most relevant descriptors are underlined and correspond to eigenvalues exceeding one…………………………………………………………………………...…..………..S12

Table S5. Eigenvalues and evolution of the percentage of information recovery in relation to the number of principal components for dataset C composed by 59 mycotoxins and 28 molecular

descriptors. The most relevant descriptors are underlined and correspond to eigenvalues greater than one……S13

Table S6. Eigenvalues and evolution of the percentage of information recovery in relation to the number of principal components for dataset E composed by 59 mycotoxins and 12 molecular descriptors. The most relevant descriptors are underlined and correspond to eigenvalues greater than one……S14

Table S7. Eigenvalues and evolution of the percentage of information recovery in relation to the number of principal components for dataset D composed by 59 mycotoxins and 39 molecular descriptors. The most relevant descriptors are underlined and correspond to eigenvalues greater than 1……S15

Table S8. Performance evaluation metrics for the LDA models. The dataset with the best performance is underlined. ……S16

Table S9. Performance evaluation metrics for the RF models. The data set with the best performance is underlined……S16

Table S10. Performance evaluation metrics for the SVM models. The dataset with the best performance is underlined……S17

Table S11. Tested parameters using Gridsearch that gave the best results in terms of accuracy of the NN models……S18

Table S12. Performance evaluation metrics for the NN models. The dataset with the best performance is underlined……S18

List of Figures

Figure S1. Silhouette plot for k-means clustering constructed over the 30 reference mycotoxin structures. The silhouette values range from -1, which means that the mycotoxins are not in the correct cluster, to +1, which means that the mycotoxin is far from the neighboring cluster and very close to the cluster to which it is assigned……S20

Figure S2. Silhouette plot for k-means clustering constructed over the 59 mycotoxins topological fingerprints. The two less cohesive clusters (light blue and light green) identified in the k-means clustering map, contain mycotoxins with a lower silhouette coefficient (0.15 and 0.21, respectively) suggesting that TENT, ENF and ATT1 are very close to the decision boundary between these neighboring groups. ENF should have been clustered with the other enniatins (pink), TENT with ergot alkaloids and ATT1 with ochratoxins. All the other groups show a good silhouette coefficient, which indicates an efficient clustering procedure……………………………………..……………………………………………S20

Figure S3. Graphical representation of the impact of molecular descriptors (loadings) on the first two principal components (correlation matrix) for the dataset composed by 30 mycotoxins. The top and the bottom plots refer to PC1 and PC2, respectively…………………………………………….S21

Figure S4. Graphical representation of the impact of molecular descriptors (loadings) on the first two principal components (correlation matrix) for dataset B composed by 59 mycotoxins and 28 molecular descriptors. The top and the bottom plots refer to PC1 and PC2, respectively…………………………………………………………………………...S22

Figure S5. Graphical representation of the impact of molecular descriptors (loadings) on the first two principal components (correlation matrix) for dataset C composed by 59 mycotoxins and 12 biological activity descriptors. The top and the bottom plots refer to PC1 and PC2, respectively…………………………………………………………………………...S23

Figure S6. Graphical representation of the impact of molecular descriptors (loadings) on the first two principal components (correlation matrix) for dataset D composed by 59 mycotoxins and 40 molecular descriptors. The top and the bottom plots refer to PC1 and PC2, respectively…………………………………………………………………………...S24

1. Evaluating molecular similarity and toxicity

Clustering results were visualized using the *Factoextra* package.7 The silhouette coefficient was used to validate K-means clustering results.8 The distance between mycotoxins were determined by the Euclidian and Tanimoto metrics.9-13 Ward's minimum variance method was applied to form groups that minimize within-group dispersion.14, 15

Biplots resulting from PCA were generated using the *FactoMineR* package of R.16 Classification analysis was performed with a training/test ratio of 0.7/0.3.17, 18 Models were built using the *MASS*19, 20 and *caret*18 packages for LDA and SVM, respectively.

The RF and NN models were trained using the Python package *Scikit Learn* (version 0.23.1). Molecular descriptors were normalized using *StandardScaler* and RF was performed using the *RandomForestClassifier* package, whereas NN was performed using *MLPClassifier*20

The GS for the design of NN was created on the basis of a systematic analysis in which the number of nodes, the maximum number of iterations, the initial learning rate controlling the step size in updating these weights, and the optimizer (*LBFGS, Stochastic Gradient Descent (SGD),* and *ADAM*) responsible for updating the weights to minimize the loss of the model were varied sequentially, as shown in Table S3, with a total of 336 combinations. The loss function was used to evaluate the NN performance and calculated as the gradient by the backpropagation algorithm.21

The confusion matrices were created and basic metrics such as accuracy, sensitivity (recall, REC, or true positive rate, TPR), specificity (true negative rate, TNR), precision (positive predictive value, PPV), and negative predictive value (NPV) calculated (as shown in Equations S1-S5, section 4).

The AUROC (Eq. S6) is a performance metric that reflects the ability of the model to discriminate between positive and negative cases.20 It is defined as the probability of a classifier to rank a true instance, True Positive value (TP), or True Negative value (TN), higher than a randomly chosen negative one22.

For the LDA and SVM models, the confusion matrix was created using the R package *caret17* and the AUROC curve was created using the package *pROC23, 24*. For RF and NN, *scikit-learn20* from Python was used to calculate these metrics.

2. Metrics extracted from confusion matrices and used to evaluate the performance of LDA, RF, SVM and NN models.

$$Accuracy = \frac{TP+TN}{TP+TN+FP+FN} \qquad \textbf{(Eq. S1)}$$

$$TPR = \frac{TP}{TP+FN} \qquad \textbf{(Eq. S2)}$$

$$TNR = \frac{TN}{FP+TN} \qquad \textbf{(Eq. S3)}$$

$$PPV = \frac{TP}{TP+FP} \qquad \textbf{(Eq. S4)}$$

$$NPV = \frac{FP}{FP+TN} \qquad \textbf{(Eq. S5)}$$

$$AUROC = \int_{-\infty}^{\infty} TPR(T)(-FPR'(T))dT \qquad \textbf{(Eq. S6)}$$

Table S1. Summary of the major features of well-known mycotoxins families.

Family	Examples	Characteristics	References
**Aflatoxins**	Aflatoxins B1, B2, G1, G2, M1<br>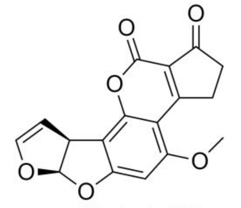<br>Aflatoxin B1	Aflatoxins are difuranocournarins derivatives and consist of a coumarin nucleus to which are attached a difuran moiety in one side and either a pentene ring or a six-membered lactone ring in the other side.	25-30
**Ergot alkaloids**	Ergotamine, Ergometrine, Ergocryptine, Ergocristine, Ergocornine, Ergosine<br>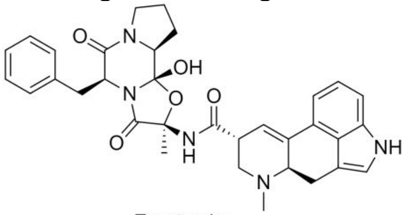<br>Ergotamine	The common structural feature of ergot alkaloids is the ergoline ring, which is methylated on the N-6 nitrogen atom, substituted on C-8, and possesses a C-8, C-9 or C-10 double bond.	31, 32
**Fumonisins**	Fumonisins A1, A2, B1, B2, B3<br>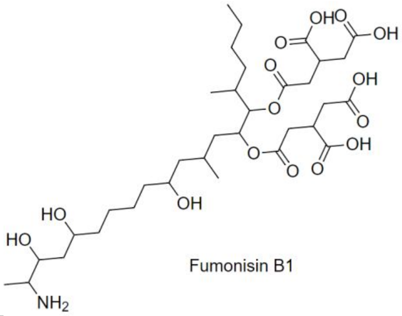<br>Fumonisin B1	Fumonisins contain 20 carbon aliphatic chain with two ester linked hydrophilic side chains. The toxic action of fumonisins is related to the competition with sphingosine in sphingolipid metabolism	33, 34
**Trichothecenes (Types A and B)**	Type A: HT-2 Toxin, T2 Toxin<br>Type B: Deoxynivalenol, Fus-X, Nivalenol<br>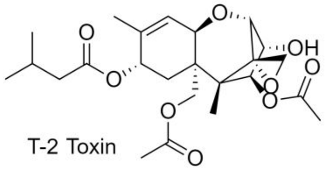<br>T-2 Toxin	Sesquiterpenoid toxins characterized by a variable number of acetoxy and hydroxyl groups, an epoxide ring at position C12-C13, and a double bond between C9 and C10.	34-37
**Patulin**	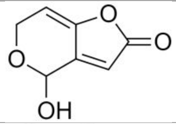	Heterocyclic lactone	38-40
**Citrinin**	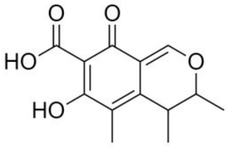	Benzopyran derivative	41, 42
**Ochratoxins**	Ochratoxins A, B, C, TC	Pentaketides consisting of a dihydro- isocoumarin	43, 44

	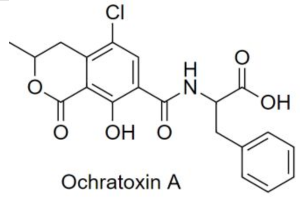 Ochratoxin A	coupled to 8-phenylalanine	
**Zearalenone**	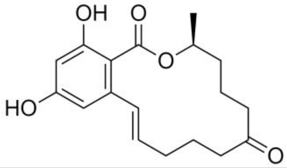	Nonsteroidal estrogen of the resorcylic acid lactone group	37, 45-47
***Alternaria* Toxins**	Altenuene, Alternariol, Alternariol methyl ether, Altertoxin, Tenuazonic acid 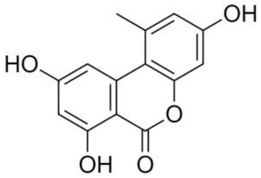 Alternariol	There are several structural types: dibenzopyrones (polyketides), perylenequinones, cyclic tetrapeptides, Anthraquinones, amine/amide metabolites and dihydroisocoumarins	48-50
**Emerging *Fusarium***	Fusaproliferin, Moliniformin, Beauvericin, NX-2 Toxin, Enniatins 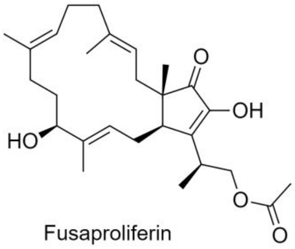 Fusaproliferin	The structure of these emerging mycotoxins can be diverse: bicyclic sesterterpene (fusaproliferin); cyclic hexadepsipeptides (ennieatins and beauvericin), organic acid (moniliformin)	47, 51, 52

Table S2. Summary of 43 molecular descriptors (15 for the reference data set and 28 for the larger data set) by category (hybrid, topological, electronic, constitutional), extracted from the CDK library after a standard feature selection procedure.

Molecular Descriptor		Description	Category	References
**Burden Chemical Abstract Service University of Texas**	BCUTw,1l	Eigenvalue based descriptor noted for its utility in chemical diversity. The descriptor is based on a weighted version of the Burden matrix which	Hybrid	[165-167]
	BCUTc,1h			

	BCUTp,1l<br>BCUTc,1l	considers both the connectivity as well as atomic properties of a molecule.<br>BCUTw,1l→ nhigh lowest atom weighted BCUTS.<br>BCUTc,1h→ nlow highest partial charge weighted BCUTS.<br>BCUTp,1l→ nhigh lowest polarizability weighted BCUTS.<br>BCUTc,1l→ nhigh lowest partial charge weighted BCUTS.		
**PetitjeanNumber**		According to the Petitjean definition, the eccentricity of a vertex corresponds to the distance from that vertex to the most remote vertex in the graph. The distance is obtained from the distance matrix as the count of edges between the two vertices. If r i is the largest matrix entry in row i of the distance matrix D, then the radius is defined as the smallest of the r i. The graph diameter D is defined as the largest vertex eccentricity in the graph. Petitjean Number is the value of diameter - radius. The radius-diameter diagram allows classification of the shapes of compounds and has remarkable properties for both graph-theoretical and geometrical shapes.	Topological	[168]
**Molecular distance-edge**	MDEC,12<br>MDEC,23<br>MDEC,33	MDE descriptors are based on two fundamental structural variables, one for distance between atoms in the molecular graph and another for edges of the adjacency in the graph.<br>Molecular distance edge:<br>MDEC,12 → between all primary and secondary carbons.<br>MDEC,23 → between all secondary and terciary carbons.<br>MDEC,33 → between all tertiary carbons.		[169,170]
	khs,dsCH			[170-172]

**Kier Hall Smarts or Eccentric Connectivity Index**	khs,dCH2<br>khs,ssO<br>khs,aaO<br>khs,sCl<br>khs,aaaC<br>khs,sNH2<br>khs,ssNH<br>khs,aaNH<br>khs,sssN	A fragment count descriptor that uses electrotopological-state fragments.<br>khs,dsCH→ [CD2H](=*)-* or =CH-<br>khs,dCH2→ [CD1H2]=* or =CH2<br>khs,ssO→ [OD2Ho](-*)-* or -O-<br>khs,aaO→ [O,oD2Ho](:*):* or :O:<br>khs,sCl→ [ClD1]-* or -Cl<br>khs,aaaC→[C,c;D3H0](:*)(:*):* or ::C:<br>khs,sNH2→ [ND1H2]-* or -NH2<br>khs,ssNH→[ND2H](-*)-* or -NH2-+<br>khs,aaNH→ [N,nD2H](:*):* or :NH:<br>khs,sssN→[ND3H0](-*)(-*)-* or >NH-+		
**Chi-chain**	VCH,5<br>SCH,5	Evaluates the simple and valence chi chain descriptors of orders 3, 4, 5, 6 and 7 by finding fragments matching SMILES strings representing the fragments corresponding to each type of chain.<br>VCH,5 → Valence chain, order 5.<br>SCH,5 → Simple chai, order 5.		[167]
**Carbon Types**	C1SP3<br>C2SP2<br>C3SP2<br>C4SP3	Characterizes de carbon connectivity in terms of hybridization.<br>C1SP3 → Singly bound carbon bound to one other carbon.<br>C2SP2 → Doubly bound carbon to two other carbons.<br>C3SP2 → Doubly bound carbon bound to three other carbons.<br>C4SP3 →Singly bound carbon to four other carbons.		[101,102,167]
**Autocorrelation Descriptor Charge**	ATSc2<br>ATSc3	Autocorrelation descriptor, weighted by charges. The values are calculated considering weight equal to charges. Explain how the values of certain functions, at intervals equal to the lag d, are correlated. In this case, lag is the topological distance, and the atomic properties (weight or charge) are the functions correlated.<br>ATSc2 → autocorrelation of a topological structure of lag 2		[170-173]

	ATSc3 → autocorrelation of a topological structure of lag 3		
**topoShape**	A measure of the anisotropy in a molecule.		[168]
**TopoPSA**	Calculation of topological polar surface area based on fragment contributions.		[168,169]
**tpsaEfficiency**	Polar surface area expressed as a ratio to molecular size.		[168,169]
**nSmallRings**	Total number of small rings of size 3 through 9.		[168,169]
**nAromRings**	Total number of small aromatic rings.		[168,169]
**nRings4**	Individual breakdown of 4 membered rings.		[168,169]
**apol**	Calculates the sum of the atomic polarizabilities, including implicit hydrogens.	Electronic	[168]
**nHBDon**	Number of hydrogen bond donors.		[168]
**nHBAcc**	Number of hydrogen bond acceptors.		[168]
**nBase**	Basic group count descriptor.		[168,169]
**MW**	Weight of atoms of a certain element type.If no element if specified, the returned value is the molecular weight.	Constitutional	[168,169]
**XlogP**	Prediction of logP based on the atom type method called XlogP.		[169]
**AlogP**	Ghose-Crippen $LogK_{ow}$		[168,169]
**Alogp2**	Square of AlogP.		[168,169]
**MLogP**	LogP based on the Mannhold equation using the number of carbons and hetero atoms.		[169]
**LipinksiFailures**	Number of failures of the Lipinksi's Rule of 5.		[169]
**nRotB**	Number of non-rotatable bonds on a molecule.		[169]

Table S3. Grid search combination parameters for the NN model. Only the best performing tested models are shown.

Architecture	Initial Learning Rate	Maximum number of iterations	Optimizer
(3,), (5,)<br>(3,3), (3,5),<br>(5,5),<br>(3,3,3), (5,5,5)	0.01<br>0.001<br>0.0005<br>0.00025	250,<br>300,<br>350,<br>400	lbfgs<br>sgd<br>adam

Table S4. Eigenvalues and data recovery evolution with respect to the number of principal components for the reference dataset composed by 30 mycotoxins and 15 molecular descriptors. The most relevant descriptors are underlined and correspond to eigenvalues exceeding 1.

	Eigenvalue	Variance %	Cumulative Variance %
**Dim. 1**	3.88	25.8	25.8
**Dim. 2**	3.14	20.9	46.8
**Dim. 3**	2.69	17.9	64.7
**Dim. 4**	1.86	12.4	77.1
**Dim. 5**	1.24	8.25	85.4
**Dim. 6**	0.818	5.46	90.8
**Dim. 7**	0.540	3.60	94.4
**Dim. 8**	0.299	1.99	96.4
**Dim. 9**	0.188	1.26	97.7
**Dim. 10**	0.139	0.926	98.6
**Dim. 11**	0.111	0.737	99.4
**Dim. 12**	0.049	0.325	99.7
**Dim. 13**	0.0278	0.185	99.9
**Dim. 14**	0.0104	0.0697	99.9
**Dim. 15**	0.00877	0.0585	100

Table S5. Eigenvalues and evolution of the percentage of information recovery in relation to the number of principal components for dataset B composed by 59 mycotoxins and 28 molecular descriptors. The most relevant descriptors are underlined and correspond to eigenvalues greater than 1.

	**Eigenvalue**	**Variance %**	**Cumulative variance %**
**Dim. 1**	5.34	19.08	19.08
**Dim. 2**	4.53	16.2	35.3
**Dim. 3**	4.28	15.3	50.5
**Dim. 4**	2.89	10.3	60.9
**Dim. 5**	2.24	8.01	68.9
**Dim. 6**	1.65	5.88	74.6
**Dim. 7**	1.52	5.42	80.2
**Dim. 8**	1.03	3.67	83.8
**Dim. 9**	0.959	3.42	87.3
**Dim. 10**	0.698	2.49	89.8
**Dim. 11**	0.605	2.16	91.9
**Dim. 12**	0.523	1.87	93.8
**Dim. 13**	0.400	1.43	95.2
**Dim. 14**	0.316	1.13	96.3
**Dim. 15**	0.260	0.931	97.3
**Dim. 16**	0.197	0.702	97.9
**Dim. 17**	0.170	0.608	98.6
**Dim. 18**	0.126	0.449	99.0
**Dim. 19**	0.0921	0.329	99.4
**Dim. 20**	0.0599	0.214	99.6
**Dim. 21**	0.0476	0.170	99.7
**Dim. 22**	0.0270	0.0965	99.8
**Dim. 23**	0.0168	0.0599	99.9
**Dim. 24**	0.0102	0.0363	99.9
**Dim. 25**	0.00804	0.0287	99.9
**Dim. 26**	0.00560	0.0200	99.9
**Dim. 27**	0.00304	0.0108	100

**Dim. 28**	2.66e-31	9.49e-31	100

Table S6. Eigenvalues and evolution of the percentage of information recovery in relation to the number of principal components for dataset D composed by 59 mycotoxins and 40 molecular descriptors. The most relevant descriptors are underlined and correspond to eigenvalues greater than 1.

	Eigenvalue	Variance %	Cumulative variance %
**Dim. 1**	11.0	28.2	28.2
**Dim. 2**	6.91	17.7	45.9
**Dim. 3**	5.07	13.0	58.9
**Dim. 4**	3.32	8.51	67.5
**Dim. 5**	2.72	6.98	74.5
**Dim. 6**	1.79	4.60	79.1
**Dim. 7**	1.64	4.19	83.3
**Dim. 8**	1.16	2.99	86.3
**Dim. 9**	1.00	2.57	88.8
**Dim. 10**	0.86	2.21	91.0
**Dim. 11**	0.63	1.61	92.7
**Dim. 12**	0.62	1.58	94.2
**Dim. 13**	0.49	1.27	95.5
**Dim. 14**	0.348	0.892	96.4
**Dim. 15**	0.325	0.833	97.2
**Dim. 16**	0.267	0.686	97.9
**Dim. 17**	0.195	0.499	98.4
**Dim. 18**	0.156	0.394	98.8
**Dim. 19**	0.114	0.292	99.1
**Dim. 20**	0.0829	0.213	99.3
**Dim. 21**	0.0749	0.192	99.5
**Dim. 22**	0.0490	0.126	99.6
**Dim. 23**	0.0349	0.0894	99.7
**Dim. 24**	0.0310	0.0796	99.8
**Dim. 25**	0.0208	0.0534	99.8

**Dim. 26**	0.0160	0.0411	99.9
**Dim. 27**	0.0109	0.0279	99.9
**Dim. 28**	0.00878	0.0225	99.9
**Dim. 29**	0.00668	0.0171	99.9
**Dim. 30**	0.00509	0.0131	99.9
**Dim. 31**	0.00383	0.00982	99.9
**Dim. 32**	0.00199	0.00511	99.9
**Dim. 33**	0.00134	0.00344	99.9
**Dim. 34**	0.00105	0.00270	99.9
**Dim. 35**	0.000457	0.00117	99.9
**Dim. 36**	0.000248	0.000637	99.9
**Dim. 37**	4.55e-06	1.17e-05	100
**Dim. 38**	7.69e-08	1.97e-07	100
**Dim. 39**	1.56e-31	4.01e-31	100

Table S7. Eigenvalues and evolution of the percentage of information recovery in relation to the number of principal components for dataset C composed by 59 mycotoxins and 12 molecular descriptors. The most relevant descriptors are underlined and correspond to eigenvalues greater than 1.

	**Eigenvalue**	**Variance %**	**Cumulative variance %**
**Dim. 1**	7.01	58.4	58.4
**Dim. 2**	2.54	21.2	79.6
**Dim. 3**	1.18	9.84	89.4
**Dim. 4**	0.729	6.08	95.5
**Dim. 5**	0.192	1.59	97.1
**Dim. 6**	0.125	1.04	98.2
**Dim. 7**	0.0741	0.618	98.8
**Dim. 8**	0.0717	0.597	99.4
**Dim. 9**	0.0319	0.267	99.6
**Dim. 10**	0.0250	0.208	99.8
**Dim. 11**	0.0154	0.128	99.9
**Dim. 12**	0.00299	0.0249	100

Table S8. Performance evaluation metrics for the LDA models. The dataset with the best performance is underlined.

	Reference	Dataset B	Dataset C	Dataset D
**Train/test: 0.7/0.3**				
**Accuracy**				
Train	0.91	0.95	0.83	0.98
Test	0.75	0.59	0.59	0.53
**Recall**				
Train	0.94	0.96	0.93	0.96
Test	0.83	0.73	0.82	0.55
**Precision**				
Train	0.94	0.96	0.84	1.00
Test	0.83	0.67	0.64	0.67
**Specificity**				
Train	0.83	0.93	0.64	1.00
Test	0.50	0.33	0.17	0.50
**AUROC**				
Train	0.89	0.95	0.79	0.98
Test	0.67	0.53	0.49	0.52

Table S9. Performance evaluation metrics for the RF models. The data set with the best performance is underlined.

	Reference	Dataset B	Dataset C	Dataset D
**Train/test: 70/30**				
**Accuracy**				
Train	1.00	1.00	1.00	1.00
Test	0.44	0.83	0.78	0.83
**Recall**				
Train	1.00	1.00	1.00	1.00
Test	0.80	1.00	0.92	1.00

**Precision**				
Train	1.00	1.00	1.00	1.00
Test	0.50	0.81	0.80	0.81
**Specificity**				
Train	1.00	1.00	1.00	1.00
Test	0.00	0.40	0.40	0.40
**AUROC**				
Train	1.00	1.00	1.00	1.00
Test	0.40	0.70	0.66	0.70

Table S10. Performance evaluation metrics for the SVM models. The dataset with the best performance is underlined.

	**Reference**	**Dataset B**	**Dataset C**	**Dataset D**
**Train/test: 70/30**				
**Accuracy**				
Train	0.86	0.90	0.74	0.90
Test	0.88	0.47	0.64	0.47
**Recall**				
Train	1.00	1.00	0.89	1.00
Test	1.00	0.64	0.91	0.64
**Precision**				
Train	0.84	0.88	0.76	0.88
Test	0.86	0.58	0.66	0.58
**Specificity**				
Train	0.50	0.71	0.43	0.71
Test	0.50	0.17	0.17	0.17
**AUROC**				
Train	0.75	0.86	0.66	0.86
Test	0.75	0.40	0.54	0.44

Table S11. Tested parameters using Gridsearch that gave the best results in terms of accuracy of the NN models.

Dataset	Best Parameters
**Reference**	Hidden layer sizes: (5,5); initial learning rate: 0.0005; maximum number of iterations: 300; solver: "adam"
**Dataset B**	Hidden layer sizes: (3,3,3); initial learning rate: 0.01; maximum number of iterations: 250; solver: "adam"
**Dataset C**	Hidden layer sizes: (3,3); initial learning rate: 0.01; maximum number of iterations: 300; solver: "sgd"
**Dataset D**	Hidden layer sizes: (3,3,3); initial learning rate: 0.001; maximum number of iterations: 250; solver: "sgd"

Table S12. Performance evaluation metrics for the NN models. The dataset with the best performance is underlined.

	Reference	Dataset B	Dataset C	Dataset D
**Train/test: 70/30**				
**Accuracy**				
Train	0.90	0.95	<u>0.73</u>	0.63
Test	0.55	0.72	<u>0.78</u>	0.72
**Recall**				
Train	1.00	0.92	<u>0.96</u>	1.00
Test	1.00	0.85	<u>0.85</u>	1.00
**Precision**				
Train	0.89	1.00	<u>0.71</u>	0.63
Test	0.56	0.79	<u>0.85</u>	0.72
**Specificity**				
Train	0.50	1.00	<u>0.33</u>	0.00
Test	0.00	0.40	<u>0.60</u>	0.00

**AUROC**				
Train	0.75	0.96	0.65	0.50
Test	0.50	0.62	0.72	0.50

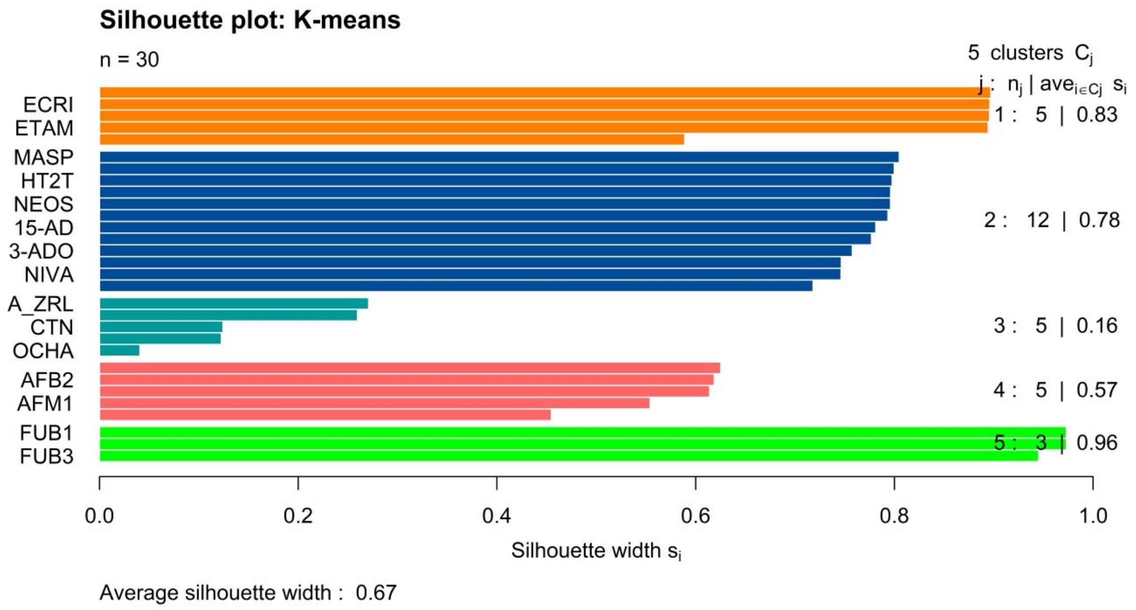

Figure S1. Silhouette plot for k-means clustering constructed over the 30 reference mycotoxin structures. The silhouette values range from -1, which means that the mycotoxins are not in the correct cluster, to +1, which means that the mycotoxin is far from the neighboring cluster and very close to the cluster to which it is assigned.

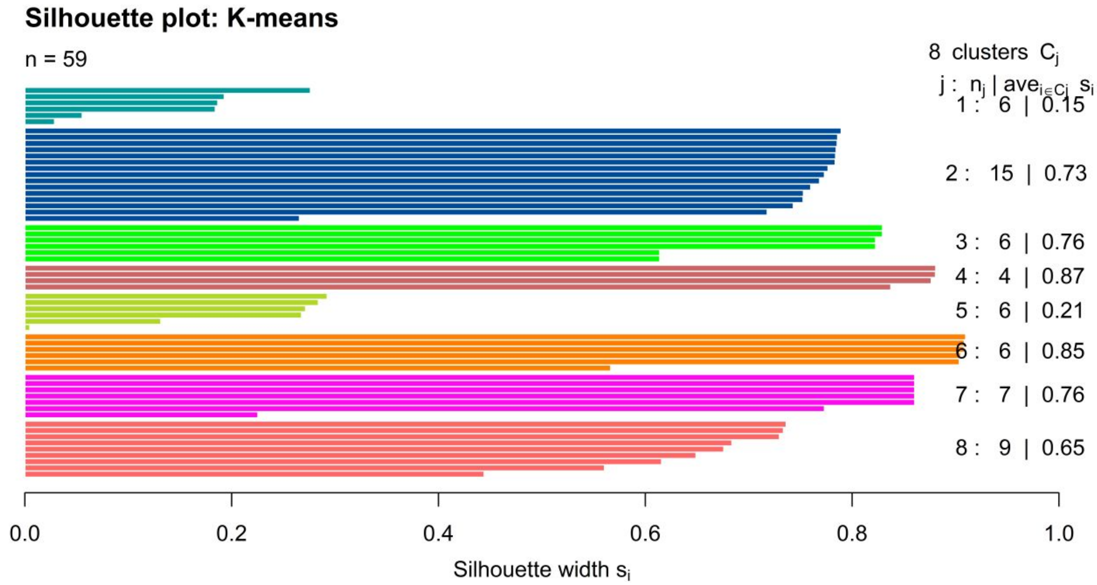

Figure S2. Silhouette plot for k-means clustering constructed over the 59 mycotoxins topological fingerprints. The two less cohesive clusters (light blue and light green) identified in the k-means clustering map, contain mycotoxins with a lower silhouette coefficient (0.15 and 0.21, respectively) suggesting that TENT, ENF and ATT1 are very close to the decision boundary between these neighboring groups. ENF should have been clustered with the other enniatins (pink), TENT with ergot alkaloids and ATT1 with ochratoxins. All the other groups show a good silhouette coefficient, which indicates an efficient clustering procedure.

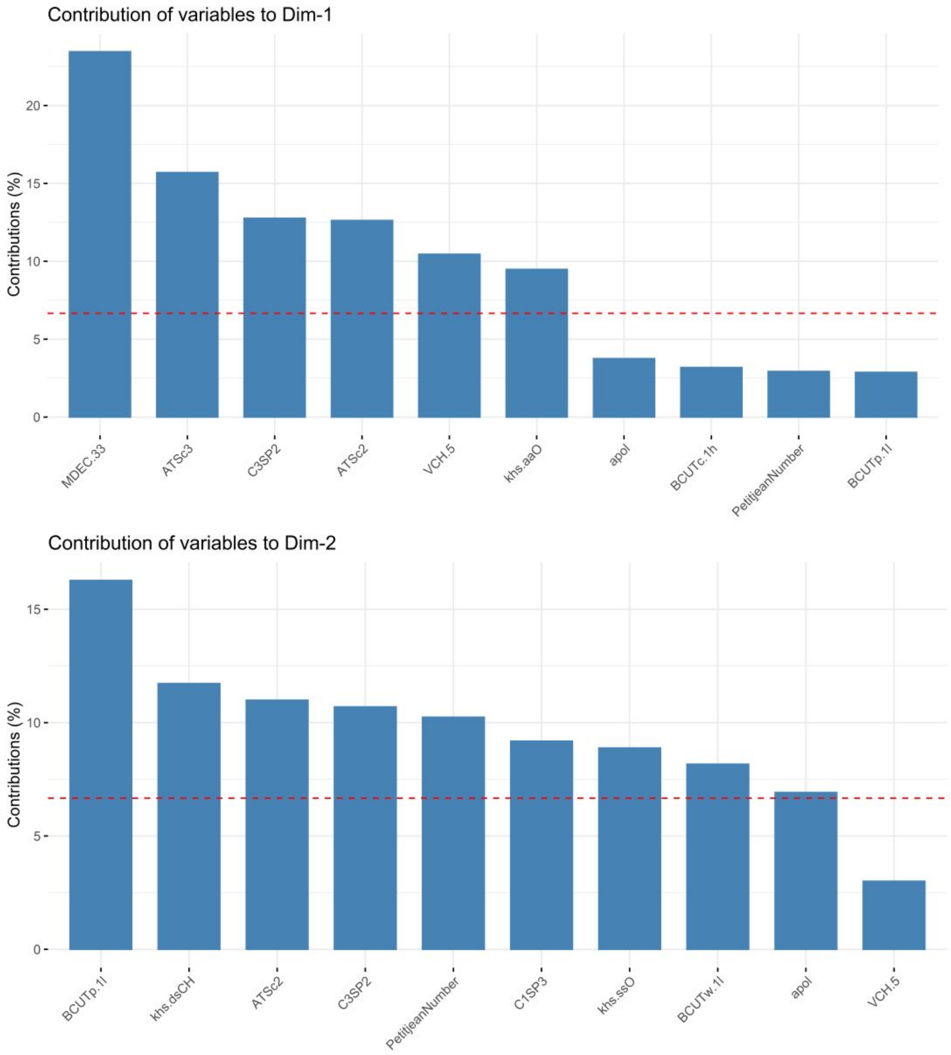

Figure S3. Graphical representation of the impact of molecular descriptors (loadings) on the first two principal components (correlation matrix) for the dataset composed by 30 mycotoxins. The top and the bottom plots refer to PC1 and PC2, respectively

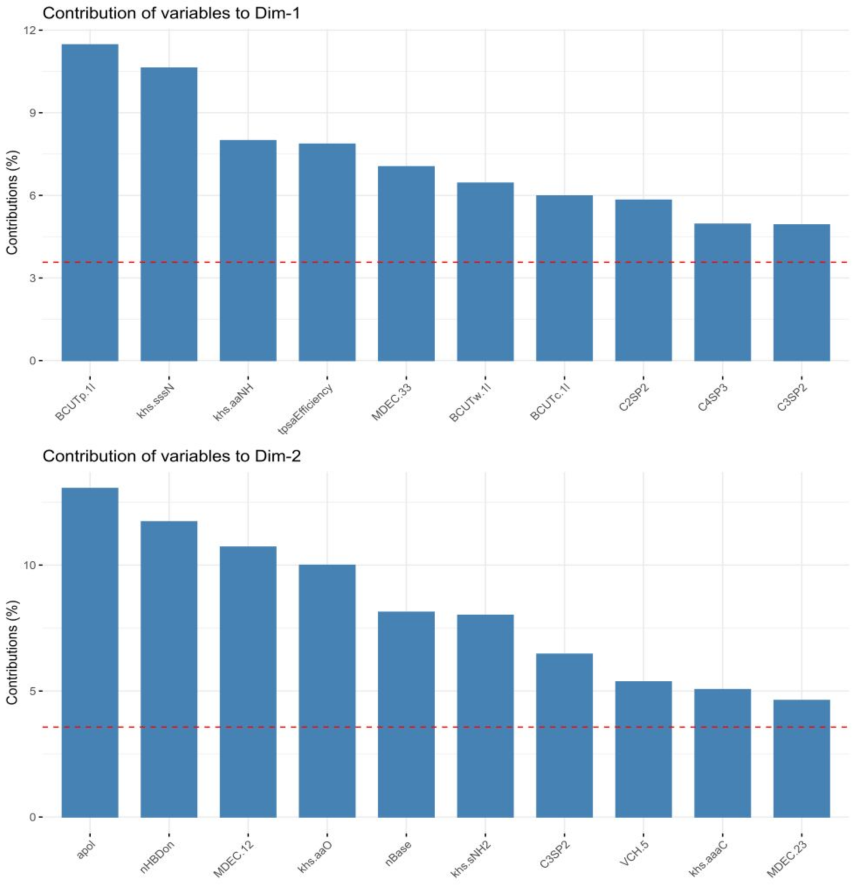

Figure S4. Graphical representation of the impact of molecular descriptors (*loadings*) on the first two principal components (correlation matrix) for dataset B composed by 59 mycotoxins and 28 molecular descriptors. The top and the bottom plots refer to PC1 and PC2, respectively.

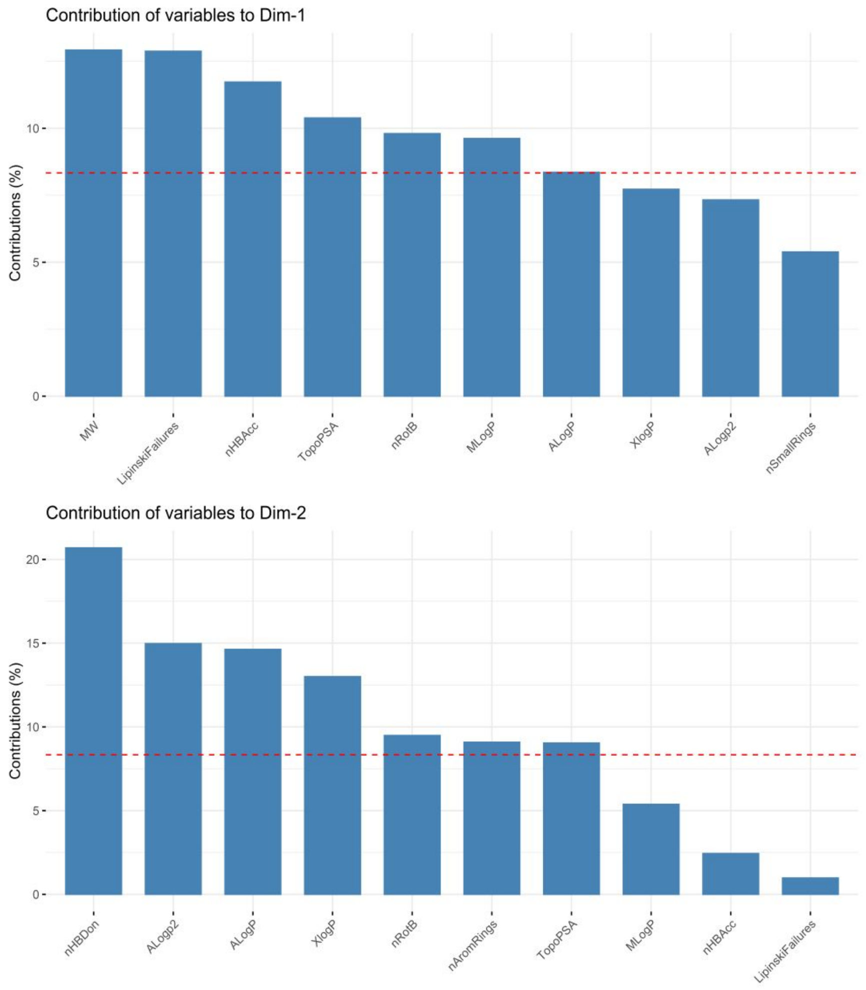

Figure S5. Graphical representation of the impact of molecular descriptors (loadings) on the first two principal components (correlation matrix) for dataset C composed by 59 mycotoxins and 12 biological activity descriptors. The top and the bottom plots refer to PC1 and PC2, respectively.

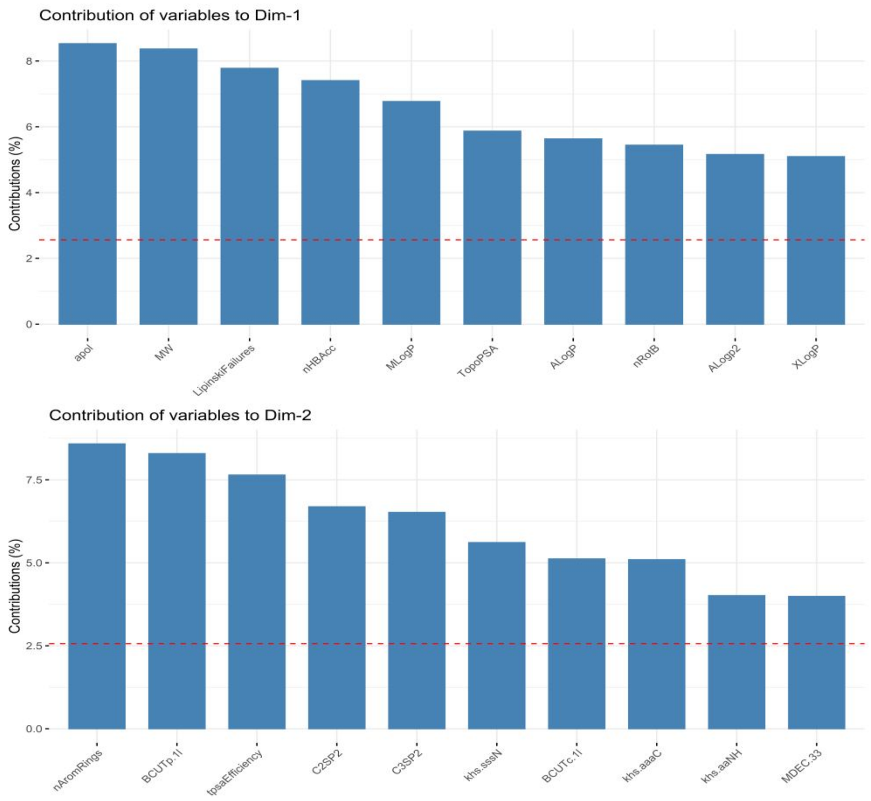

Figure S6. Graphical representation of the impact of molecular descriptors (*loadings*) on the first two principal components (correlation matrix) for dataset D composed by 59 mycotoxins and 40 molecular descriptors. The top and the bottom plots refer to PC1 and PC2, respectively.

References

1. Cao, D. S.; Xu, Q. S.; Hu, Q. N.; Liang, Y. Z., ChemoPy: freely available python package for computational biology and chemoinformatics. *Bioinformatics* **2013,** *29* (8), 1092-4.
2. Guha, R., Chemical Informatics Functionality in R. *Journal of Statistical Software* **2007,** *18* (5), 1 - 16.
3. Willighagen, E. L.; Mayfield, J. W.; Alvarsson, J.; Berg, A.; Carlsson, L.; Jeliazkova, N.; Kuhn, S.; Pluskal, T.; Rojas-Chertó, M.; Spjuth, O.; Torrance, G.; Evelo, C. T.; Guha, R.;

Steinbeck, C., The Chemistry Development Kit (CDK) v2.0: atom typing, depiction, molecular formulas, and substructure searching. *Journal of Cheminformatics* **2017,** *9* (1), 33.

4. Steinbeck, C.; Han, Y.; Kuhn, S.; Horlacher, O.; Luttmann, E.; Willighagen, E., The Chemistry Development Kit (CDK): an open-source Java library for Chemo- and Bioinformatics. *J Chem Inf Comput Sci* **2003,** *43* (2), 493-500.
5. Guha, R.; Cherto, M. R., rcdk: Integrating the CDK with R. CRAN: 2017.
6. Maechler, M.; Rousseeuw, P.; Struyf, A.; Hubert, M.; Hornik, K., Cluster: cluster analysis basics and extensions. *R package version* **2012,** *1* (2), 56.
7. Kassambara, A.; Mundt, F., Package factoextra: Extract and visualize the Results of Multivariate Data Analyses. R Package Version 1.0. 7. 2017.
8. Kassambara, A., *Practical guide to cluster analysis in R: Unsupervised machine learning*. Sthda: 2017; Vol. 1.
9. Vamathevan, J.; Clark, D.; Czodrowski, P.; Dunham, I.; Ferran, E.; Lee, G.; Li, B.; Madabhushi, A.; Shah, P.; Spitzer, M., Applications of machine learning in drug discovery and development. *Nature reviews Drug discovery* **2019,** *18* (6), 463-477.
10. Bajorath, J., Selected Concepts and Investigations in Compound Classification, Molecular Descriptor Analysis, and Virtual Screening. *Journal of Chemical Information and Computer Sciences* **2001,** *41* (2), 233-245.
11. Cereto-Massagué, A.; Ojeda, M. J.; Valls, C.; Mulero, M.; Garcia-Vallvé, S.; Pujadas, G., Molecular fingerprint similarity search in virtual screening. *Methods* **2015,** *71*, 58-63.
12. Raymond, J. W.; Willett, P., Effectiveness of graph-based and fingerprint-based similarity measures for virtual screening of 2D chemical structure databases. *J Comput Aided Mol Des* **2002,** *16* (1), 59-71.
13. Lo, Y.-C.; Rensi, S. E.; Torng, W.; Altman, R. B., Machine learning in chemoinformatics and drug discovery. *Drug Discovery Today* **2018,** *23* (8), 1538-1546.
14. Cova, T. F. G. G.; Pereira, J. L. G. F. S. C.; Pais, A. A. C. C., Is standard multivariate analysis sufficient in clinical and epidemiological studies? *Journal of Biomedical Informatics* **2013,** *46* (1), 75-86.
15. Murtagh, F.; Legendre, P., Ward's Hierarchical Agglomerative Clustering Method: Which Algorithms Implement Ward's Criterion? *Journal of Classification* **2014,** *31* (3), 274-295.
16. Husson, F.; Josse, J.; Le, S.; Mazet, J.; Husson, M. F., Package 'factominer'. *An R package* **2016,** *96*, 698.
17. Kuhn, M.; Wing, J.; Weston, S.; Williams, A., The caret package. *Gene Expr* **2007**.
18. Kuhn, M., Building predictive models in R using the caret package. *Journal of statistical software* **2008,** *28*, 1-26.
19. Ripley, B.; Venables, B.; Bates, D. M.; Hornik, K.; Gebhardt, A.; Firth, D.; Ripley, M. B., Package 'mass'. *Cran r* **2013,** *538*, 113-120.
20. Pedregosa, F.; Varoquaux, G.; Gramfort, A.; Michel, V.; Thirion, B.; Grisel, O.; Blondel, M.; Prettenhofer, P.; Weiss, R.; Dubourg, V., Scikit-learn: Machine learning in Python. *the Journal of machine Learning research* **2011,** *12*, 2825-2830.
21. Karlsson, L.; Bonde, O., A comparison of selected optimization methods for neural networks. 2020.
22. Beauxis-Aussalet, E.; Hardman, L. In *Visualization of confusion matrix for non-expert users*, IEEE Conference on Visual Analytics Science and Technology (VAST)-Poster Proceedings, 2014.
23. Benkerroum, N., Aflatoxins: Producing-Molds, Structure, Health Issues and Incidence in Southeast Asian and Sub-Saharan African Countries. *Int J Environ Res Public Health* **2020,** *17* (4).
24. Iqbal, S. Z., Mycotoxins in food, recent development in food analysis and future challenges; a review. *Current Opinion in Food Science* **2021,** *42*, 1-+.
25. Juraschek, L. M.; Kappenberg, A.; Amelung, W., Mycotoxins in soil and environment. *Sci Total Environ* **2022,** *814*, 152425.

26. Kumar, P.; Mahato, D. K.; Kamle, M.; Mohanta, T. K.; Kang, S. G., Aflatoxins: A Global Concern for Food Safety, Human Health and Their Management. *Frontiers in Microbiology* **2017,** *7*, 2170.
27. Udomkun, P.; Wiredu, A. N.; Nagle, M.; Muller, J.; Vanlauwe, B.; Bandyopadhyay, R., Innovative technologies to manage aflatoxins in foods and feeds and the profitability of application - A review. *Food Control* **2017,** *76*, 127-138.
28. Frisvad, J. C.; Hubka, V.; Ezekiel, C.; Hong, S.-B.; Nováková, A.; Chen, A.; Arzanlou, M.; Larsen, T.; Sklenář, F.; Mahakarnchanakul, W., Taxonomy of Aspergillus section Flavi and their production of aflatoxins, ochratoxins and other mycotoxins. *Studies in mycology* **2018,** *91* (1), 37-59.
29. Krska, R.; Crews, C., Significance, chemistry and determination of ergot alkaloids: a review. *Food Addit Contam Part A Chem Anal Control Expo Risk Assess* **2008,** *25* (6), 722-31.
30. Gerhards, N.; Matuschek, M.; Wallwey, C.; Li, S. M., Genome mining of ascomycetous fungi reveals their genetic potential for ergot alkaloid production. *Arch Microbiol* **2015,** *197* (5), 701-13.
31. de Matos, N. A. V.; de Moraes, M. H. P.; Sartori, A. V.; do Couto Jacob, S., Optimization and Validation of an Analytical Method for the Determination of Free and Hidden Fumonisins in Corn and Corn Products by UHPLC-MS/MS. *Food Analytical Methods* **2021,** *14* (8), 1611-1624.
32. Schaarschmidt, S.; Fauhl-Hassek, C., The fate of mycotoxins during the primary food processing of maize. *Food Control* **2021,** *121*, 107651.
33. McCormick, S. P.; Stanley, A. M.; Stover, N. A.; Alexander, N. J., Trichothecenes: from simple to complex mycotoxins. *Toxins (Basel)* **2011,** *3* (7), 802-14.
34. Wang, W. J.; Zhu, Y.; Abraham, N.; Li, X. Z.; Kimber, M.; Zhou, T., The Ribosome-Binding Mode of Trichothecene Mycotoxins Rationalizes Their Structure-Activity Relationships. *International Journal of Molecular Sciences* **2021,** *22* (4), 1604.
35. Pack, E. D.; Weiland, S.; Musser, R.; Schmale, D. G., Survey of zearalenone and type-B trichothecene mycotoxins in swine feed in the USA. *Mycotoxin Research* **2021,** *37* (4), 297-313.
36. Chen, H.; Cao, L.; Han, K.; Zhang, H.; Cui, J.; Ma, X.; Zhao, S.; Zhao, C.; Yin, S.; Fan, L.; Hu, H., Patulin disrupts SLC7A11-cystine-cysteine-GSH antioxidant system and promotes renal cell ferroptosis both in vitro and in vivo. *Food Chem Toxicol* **2022,** *166*, 113255.
37. Iqbal, S. Z.; Akbar, M.; Razis, A. F. A.; Waqas, M., Assessment of patulin in different cultivars of apples, juices, and distribution in decay portion. *International Journal of Environmental Analytical Chemistry* **2022**, 1-11.
38. Skoko, A. G.; Vilic, R.; Kovac, M.; Nevistic, A.; Sarkanj, B.; Lores, M.; Celeiro, M.; Babojelic, M. S.; Kovac, T.; Loncaric, A., Occurrence of Patulin and Polyphenol Profile of Croatian Traditional and Conventional Apple Cultivars during Storage. *Foods* **2022,** *11* (13), 1912.
39. Wu, S. W.; Ko, J. L.; Liu, B. H.; Yu, F. Y., Pilot production of a sensitive ELISA kit and an immunochromatographic strip for rapid detecting citrinin in fermented rice. *RSC Adv* **2022,** *12* (31), 19981-19989.
40. Kamle, M.; Mahato, D. K.; Gupta, A.; Pandhi, S.; Sharma, N.; Sharma, B.; Mishra, S.; Arora, S.; Selvakumar, R.; Saurabh, V.; Dhakane-Lad, J.; Kumar, M.; Barua, S.; Kumar, A.; Gamlath, S.; Kumar, P., Citrinin Mycotoxin Contamination in Food and Feed: Impact on Agriculture, Human Health, and Detection and Management Strategies. *Toxins (Basel)* **2022,** *14* (2), 85.
41. Reddy, L.; Bhoola, K., Ochratoxins-food contaminants: impact on human health. *Toxins (Basel)* **2010,** *2* (4), 771-9.
42. Li, S.; Kang, Y.; Shang, M. D.; Cai, Y. F.; Yang, Z. Q., Highly sensitive and selective detection of Ochratoxin a using modified graphene oxide-aptamer sensors as well as application. *Microchemical Journal* **2022,** *179*, 107449.
43. Ning, H.; Wang, J.; Jiang, H.; Chen, Q., Quantitative detection of zearalenone in wheat grains based on near-infrared spectroscopy. *Spectrochim Acta A Mol Biomol Spectrosc* **2022,** *280*, 121545.

44. Feng, Y. Q.; Zhao, A. H.; Wang, J. J.; Tian, Y.; Yan, Z. H.; Dri, M.; Shen, W.; De Felici, M.; Li, L., Oxidative stress as a plausible mechanism for zearalenone to induce genome toxicity. *Gene* **2022,** *829*, 146511.
45. Bai, J.; Zhou, Y.; Luo, X.; Hai, J.; Si, X.; Li, J.; Fu, H.; Dai, Z.; Yang, Y.; Wu, Z., Roles of stress response-related signaling and its contribution to the toxicity of zearalenone in mammals. *Comprehensive Reviews in Food Science and Food Safety* **2022,** *21* (4), 3326-3345.
46. Ji, X. F.; Xiao, Y. P.; Jin, C. H.; Wang, W.; Lyu, W. T.; Tang, B.; Yang, H., Alternaria mycotoxins in food commodities marketed through e-commerce stores in China: Occurrence and risk assessment. *Food Control* **2022,** *140*, 109125.
47. Aichinger, G.; Del Favero, G.; Warth, B.; Marko, D., Alternaria toxins-Still emerging? *Compr Rev Food Sci Food Saf* **2021,** *20* (5), 4390-4406.
48. Chen, A.; Mao, X.; Sun, Q.; Wei, Z.; Li, J.; You, Y.; Zhao, J.; Jiang, G.; Wu, Y.; Wang, L.; Li, Y., Alternaria Mycotoxins: An Overview of Toxicity, Metabolism, and Analysis in Food. *Journal of Agricultural and Food Chemistry* **2021,** *69* (28), 7817-7830.
49. Sa, S. V. M.; Monteiro, C.; Fernandes, J. O.; Pinto, E.; Faria, M. A.; Cunha, S. C., Emerging mycotoxins in infant and children foods: A review. *Crit Rev Food Sci Nutr* **2021**, 1-15.
50. Ekwomadu, T. I.; Akinola, S. A.; Mwanza, M., Fusarium Mycotoxins, Their Metabolites (Free, Emerging, and Masked), Food Safety Concerns, and Health Impacts. *International Journal of Environmental Research and Public Health* **2021,** *18* (22), 11741.
